# Host Pah1p phosphatidate phosphatase limits viral replication by regulating phospholipid synthesis

**DOI:** 10.1371/journal.ppat.1006988

**Published:** 2018-04-12

**Authors:** Zhenlu Zhang, Guijuan He, Gil-Soo Han, Jiantao Zhang, Nicholas Catanzaro, Arturo Diaz, Zujian Wu, George M. Carman, Lianhui Xie, Xiaofeng Wang

**Affiliations:** 1 Fujian Province Key Laboratory of Plant Virology, Institute of Plant Virology, Fujian Agriculture and Forestry University, Fuzhou, Fujian, P. R. China; 2 Department of Plant Pathology, Physiology, and Weed Science, Virginia Tech, Blacksburg, VA, United States of America; 3 Department of Food Science and the Rutgers Center for Lipid Research, New Jersey Institute for Food, Nutrition, and Health, Rutgers University, New Brunswick, NJ, United States of America; 4 Department of Biomedical Sciences and Pathobiology, Virginia-Maryland College of Veterinary Medicine, Virginia Tech, Blacksburg, VA, United States of America; 5 Department of Biology, La Sierra University, Riverside, VA, United States of America; Agriculture and Agri-Food Canada, CANADA

## Abstract

Replication of positive-strand RNA viruses [(+)RNA viruses] takes place in membrane-bound viral replication complexes (VRCs). Formation of VRCs requires virus-mediated manipulation of cellular lipid synthesis. Here, we report significantly enhanced brome mosaic virus (BMV) replication and much improved cell growth in yeast cells lacking *PAH1* (*pah1*Δ), the sole yeast ortholog of human *LIPIN* genes. *PAH1* encodes Pah1p (phosphatidic acid phosphohydrolase), which converts phosphatidate (PA) to diacylglycerol that is subsequently used for the synthesis of the storage lipid triacylglycerol. Inactivation of Pah1p leads to altered lipid composition, including high levels of PA, total phospholipids, ergosterol ester, and free fatty acids, as well as expansion of the nuclear membrane. In *pah1*Δ cells, BMV replication protein 1a and double-stranded RNA localized to the extended nuclear membrane, there was a significant increase in the number of VRCs formed, and BMV genomic replication increased by 2-fold compared to wild-type cells. In another yeast mutant that lacks both *PAH1* and *DGK1* (encodes diacylglycerol kinase converting diacylglycerol to PA), which has a normal nuclear membrane but maintains similar lipid compositional changes as in *pah1*Δ cells, BMV replicated as efficiently as in *pah1*Δ cells, suggesting that the altered lipid composition was responsible for the enhanced BMV replication. We further showed that increased levels of total phospholipids play an important role because the enhanced BMV replication required active synthesis of phosphatidylcholine, the major membrane phospholipid. Moreover, overexpression of a phosphatidylcholine synthesis gene (*CHO2*) promoted BMV replication. Conversely, overexpression of *PAH1* or plant *PAH1* orthologs inhibited BMV replication in yeast or *Nicotiana benthamiana* plants. Competing with its host for limited resources, BMV inhibited host growth, which was markedly alleviated in *pah1*Δ cells. Our work suggests that Pah1p promotes storage lipid synthesis and thus represses phospholipid synthesis, which in turn restricts both viral replication and cell growth during viral infection.

## Author summary

Phosphatidate (PA) plays crucial roles in lipid metabolism because it is the shared precursor for major membrane component phospholipids and for storage lipid triacylglycerols (TAGs). Phosphatidate phosphatase (PAP) is involved in converting PA to TAG via diacylglycerol and directs the lipid flux from membrane synthesis for cell growth to lipid storage during inactive growth stages. PAP is structurally and functionally conserved among eukaryotes, from Pah1p in yeast to lipins in mammals. Inactivation of PAP substantially affects lipid composition and causes human diseases, such as lipodystrophy and insulin resistance, whereas overexpression of PAP leads to obesity. We show that viruses compete with host cells for limited lipids, inhibiting host growth as a consequence. In yeast cells with inactive PAP, total phospholipids, sterol esters, and free fatty acids accumulated to high levels at the expense of TAG. Altered lipid composition substantially promotes cell growth during viral replication. However, this simultaneously enhances genomic replication of brome mosaic virus (BMV), a well-studied model for examining positive-strand RNA viruses. These results suggest that PAP restricts virus replication by limiting phospholipid synthesis, which is required for viral replication. Our data strengthen the notion that viruses may take advantage of disrupted PAP or other nutrient homeostasis for infection.

## Introduction

Positive-strand RNA viruses [(+)RNA viruses] are the largest of all virus classes and cause numerous important diseases in humans, animals, and plants. All of the well-studied (+)RNA viruses have been shown to remodel host intracellular membranes to build viral replication complexes (VRCs) for genomic replication [1–4]. Because cellular lipids are the major building blocks of membranes, their metabolism and/or composition are crucial for virus-induced membrane rearrangements [4–6].

Brome mosaic virus (BMV) is the type member of the family *Bromoviridae* and a representative member of the alphavirus-like superfamily [7]. BMV induces spherular VRCs at the perinuclear endoplasmic reticulum (nER) membrane in the yeast *Saccharomyces cerevisiae* and in barley cells [8–11]. BMV has three capped genomic RNAs and a subgenomic mRNA, RNA4. For viral replication, RNA1- and RNA2-encoded replication proteins 1a and 2a polymerase (2a^pol^) are necessary and sufficient for BMV replication in barley and *Nicotiana benthamiana* [7,12,13] as well as in yeast [11]. With a central RNA-dependent RNA polymerase (RdRp) domain, 2a^pol^ serves as the replicase. In addition, the N-terminus of 2a^pol^ interacts with the C-terminal domain of 1a [14–16]. 1a has an N-terminal RNA capping domain that adds a cap to the 5’ end of viral RNAs [17–19] and a C-terminal ATPase/helicase-like domain that is required for translocating viral genomic RNAs into VRCs [20]. 1a localizes to the nER membrane, which is the nuclear membrane or nuclear envelop, where it invaginates the outer nER membrane into the ER lumen to form spherules that have an overall negative membrane curvature [11,21]. Spherules become VRCs when 2a^pol^ and viral genomic RNAs are recruited by 1a during viral replication [11]. Several properties of 1a are required for this process, including its membrane association domain, an amphipathic α-helix (1a amino acids 392–407) [22], and its ability to self-interact [23].

**Fig 1:**
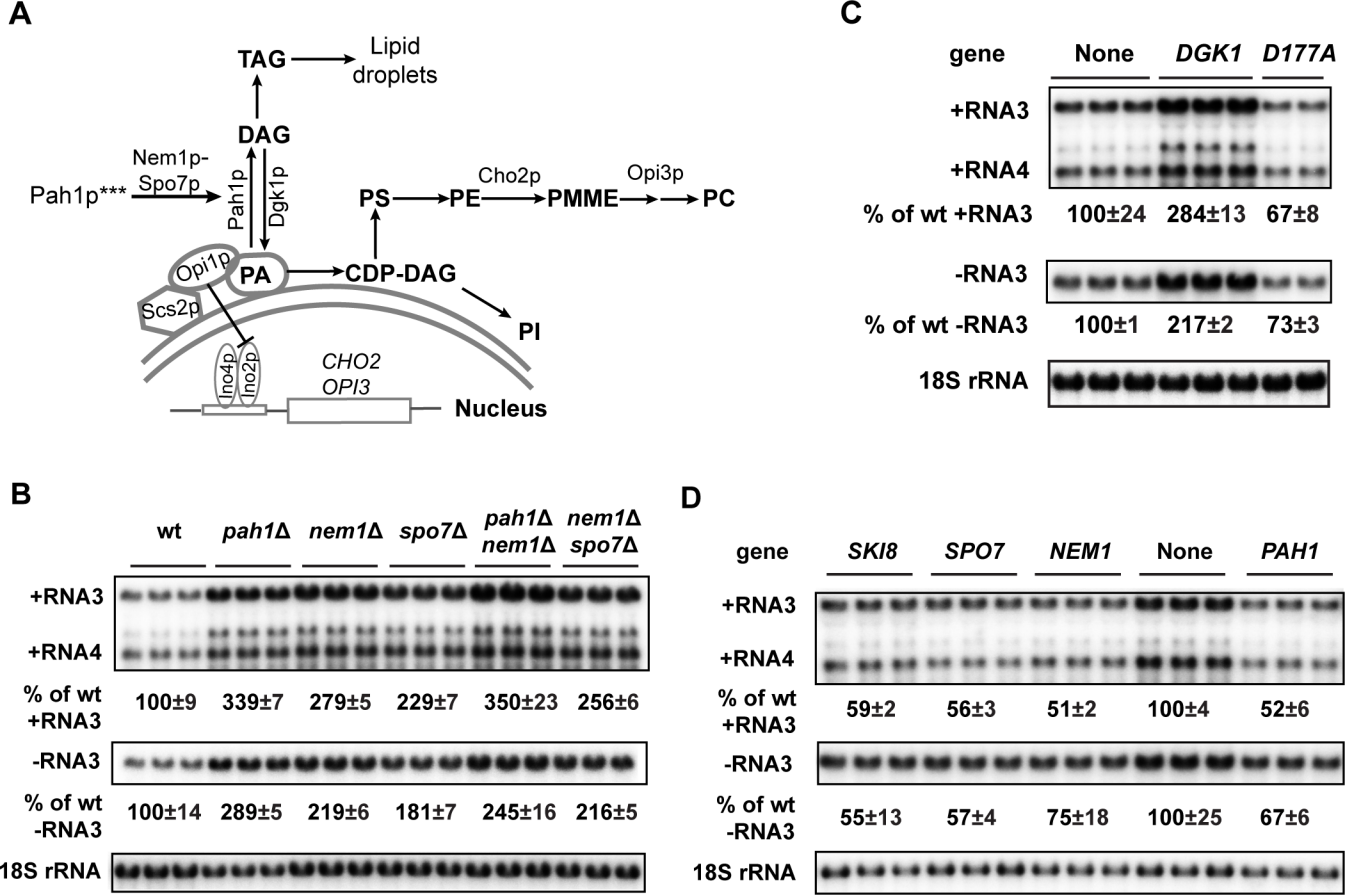
The inactivation or disruption of Pah1p promotes BMV genomic replication. (A) Diagram of lipid metabolism in yeast. Key enzymes are shown. PA serves as a substrate for phospholipids and TAG. PA and Scs2p bind to and sequester Opi1p, keeping it from reaching to the nucleus, where Opi1p interacts with Ino2p and represses transcription of *CHO2*, *OPI3* and other genes involved in phospholipid synthesis. Pah1p*** represents the hyperphosphorylated inactive Pah1p. (B) Accumulated BMV RNAs in wt and mutant cells with *PAH1* deleted or Pah1p inactivated. Positive- and negative-strand viral RNAs were detected by using BMV RNA strand-specific probes. 18S rRNA was included as a control to eliminate loading variations. All experiments shown in the figure and in subsequent figures have been repeated multiple times and a representative figure is shown. (C) BMV replication in wt cells overexpressing wt or a defective mutant *D177A* of Dgk1p. (D) BMV replication in wt cells overexpressing *PAH1*, *NEM1*, *SPO7*, or *SKI8*. *SKI8*, a well-known antiviral gene, serves as a positive control.

Lipids play crucial roles in BMV replication, similar to other (+)RNA viruses [5,6]. In yeast, an ~30% increase of accumulated total fatty acids (FAs) per cell was induced by the expression of 1a along with the formation of spherules [24]. A mild decrease in unsaturated FAs (UFAs) inhibited BMV RNA replication more than 20-fold [24,25]. It was further shown that the decreased UFAs particularly affected the membranes surrounding VRCs, indicating that the lipid environment of VRC membranes is different from the rest of the nER membrane [24,25]. BMV replication also requires host *ACB1*-encoded acyl-Coenzyme A (acyl-CoA) binding protein, which binds to long-chain fatty acyl-CoAs and is important in maintaining lipid homeostasis. In the *ACB1* deletion mutant, BMV RNA replication is inhibited by more than 10-fold and spherules are smaller in size but greater in number than those in wild-type (wt) cells [26]. Enhanced accumulation of phosphatidylcholine (PC) is also associated with BMV replication sites [27]. In addition, cellular PC synthesis enzyme Cho2p (phosphatidylethanolamine (PE) methyltransferase) (Fig 1A) is recruited to BMV replication sites by 1a via a specific 1a-Cho2p interaction, suggesting an enhanced PC synthesis at the viral replication sites. As expected, deletion of *CHO2* significantly inhibits BMV replication, raising the possibility of controlling the viral replication by blocking the 1a-mediated Cho2p recruitment [27].

Phosphatidate (PA) is a common precursor for both phospholipids and storage lipids. PA is produced *de novo* from glycerol-3-phosphate [28–30] and can be converted to CDP-diacylglycerol (CDP-DAG) [31–33], which is subsequently used to produce phospholipids, including PC, PE, phosphatidylinositol (PI), and phosphatidylserine (PS) (Fig 1A). PA can also be converted to diacylglycerol (DAG) by *PAH1*-encoded Pah1p, which is an Mg^2+^-dependent phosphatidate phosphatase, and further to TAG, the major storage lipid [34]. In yeast, PA also regulates the expression of lipid synthesis genes by sequestering a transcription repressor, Opi1p (overproduction of inositol1), at the nER membrane [35]. When PA levels are low, Opi1p is released from the nuclear membranes and translocated to the nucleus to repress transcriptions of many genes involved in phospholipid synthesis, including *CHO2* and *OPI3* (Fig 1A) [35,36].

Pah1p is highly regulated given its important roles in directing PA for the synthesis of storage lipids and thus, away from phospholipid synthesis [37–39]. Primarily localized in the cytosol as a hyperphosphorylated inactive form, Pah1p is dephosphorylated by a phosphatase complex that is composed of the catalytic subunit Nem1p (nuclear envelop morphology1) and the regulatory partner Spo7p (sporulation7) [40–42]. The Nem1p-Spo7p complex also recruits Pah1p to ER membranes where the active Pah1p is associated with membranes via an insertion of an amphipathic α-helix [43,44]. Both Nem1p and Spo7p are required for protein phosphatase activity and absence of either subunit inactivates the protein phosphatase activity of the complex, and thus, Pah1p PA phosphatase activity [42]. In *PAH1* deletion mutant (*pah1*Δ) cells, total phospholipid levels increase by ~2-fold while TAG levels decrease significantly and in addition, the nER membrane expanded compared to that of wt cells [40,45]. It has been shown that in *pah1*Δ cells, the nER membrane always expands at the site close to nucleolus, the site of ribosome biogenesis, and the chromosome DNA-occupied area remain the same as that in wt cells [46]. Pah1p shares structural and functional similarities to human lipins (lipin1, 2, and 3) as well as to AtPah1 and AtPah2 of *Arabidopsis thaliana* in that *LIPINs* or *AtPAHs* can complement phenotypical defects in yeast *pah1*Δ cells, including the decreased TAG and expanded nER membrane [47–49].

In a previous large-scale screening of a yeast deletion array, it was found that deleting *NEM1* or *SPO7* significantly enhanced BMV replication [50]. In addition, deletion of *PAH1* facilitates robust RNA replication of tomato bushy stunt virus (TBSV). TBSV normally replicates in peroxisomes but assemble their VRCs at expanded ER membranes in *pah1*Δ cells. In addition, TBSV VRCs in *pah1Δ* cells are more active than those in wt cells [51,52].

Here, we report that disruption of *PAH1* promotes BMV replication and results in the formation of VRCs that are 2-fold more abundant in number compared to those in wt cells, suggesting that a group of (+)RNA viruses could take advantage of the inactivation of Pah1p to promote their replication. We further demonstrate that the enhanced BMV replication phenotype is not due to the extended nER membrane but due to the increase in total phospholipid levels. In addition, we show that deleting *PAH1* also alleviates BMV-inhibited yeast cell growth. We conclude that Pah1p, by targeting lipid flux away from phospholipid synthesis, constrains both viral replication and cell growth during BMV replication.

## Results

### There is a direct correlation between the inactivation of Pah1p and enhanced BMV RNA replication in yeast

In a previous genome-wide screen of yeast deletion mutants in which BMV RNA replication was measured by the expression of a *Renilla* luciferase reporter, there was a dramatic increase in BMV replication in yeast strains that had either *NEM1* or *SPO7* deleted [50]. The *pah1*Δ mutant, however, was missing from the library when the screen was performed. Since both Nem1p and Spo7p are required for Pah1p activation, deleting *NEM1*, *SPO7* or *PAH1* causes similar phenotypes in yeast [40,53]. To validate results of the screen and to determine the possible role of Pah1p in BMV replication, we tested BMV replication in *nem1*Δ, *spo7*Δ, and *pah1*Δ single mutants, as well as the double mutants *pah1*Δ *nem1*Δ and *nem1*Δ *spo7*Δ by performing Northern hybridization with viral RNA strand-specific probes. As shown in Fig 1B, in the *nem1*Δ or *spo7*Δ mutants, both negative- and positive-strand RNA accumulation increased by approximately 2-fold compared to those in wt cells. However, no further increase of BMV RNA replication was observed when both *NEM1* and *SPO7* were deleted, agreeing well with the notion that each is necessary to activate Pah1p. Providing further support that Pah1p restricts BMV replication, deleting *PAH1* enhanced BMV positive- and negative-strand RNA3 accumulation by about 3-fold compared to that in wt cells (Fig 1B). It should be noted, however, that cells in which both *PAH1* and *NEM1* were deleted consistently supported the highest levels of BMV genomic replication; thus, we used this double mutant in the majority of experiments described below (Fig 1B). These data indicated that a lack of, or inactivation of, Pah1p promoted BMV replication in yeast, most likely through the increased production of PA and thus, increased total phospholipids and the expanded nER membrane.

To strengthen the notion that increased PA levels in *pah1*Δ cells is a major contributor to the enhanced BMV replication, we tested whether BMV replication was affected by overexpressing *DGK1*. *DGK1* encodes DAG kinase, which converts DAG to PA in yeast (Fig 1A). Similar to deleting *PAH1*, overexpressing *DGK1* leads to a decrease in TAG accumulation and an increase in PA levels, resulting in an expanded nER membrane in yeast cells [54]. As expected, overexpression of *DGK1* also enhanced BMV RNA replication to levels comparable to that in *pah1*Δ cells (Fig 1C). To confirm that Dgk1p enzymatic activity was required for the effect, we used a Dgk1p mutant, *D177A*, which lacks DAG kinase activity and whose overexpression does not extend the nER membrane [54]. Indeed, overexpression of *D177A* did not promote BMV RNA replication (Fig 1C), consistent with the notion that redirecting lipid synthesis from TAG to phospholipids could enhance BMV replication.

In contrast to the above deletion mutants, overexpression of *PAH1* inhibited BMV replication ~2-fold (Fig 1D). Similar inhibition in BMV replication was also observed in yeast cells overexpressing *NEM1* or *SPO7*. These effects were comparable to that of *SKI8* (superkiller8), a well-known antiviral gene [50,55] (Fig 1D). Taken together, our results indicate that there is a positive correlation between the inactivation or disruption of Pah1p function and enhanced BMV replication levels in yeast, indicating that Pah1p is a limiting factor for BMV replication.

### BMV 1a localizes to the extended nuclear membrane in cells lacking *PAH1*

A dramatically extended nER membrane is present in cells lacking *NEM1*, *SPO7*, and/or *PAH1* [40,53] or when *DGK1* is overexpressed [54]. Since BMV 1a invaginates the outer nER membrane into the lumen to form spherules, the extended nER membrane in these mutant cells may provide an expanded surface area for VRC formation and thus, promote BMV replication [11].

**Fig 2:**
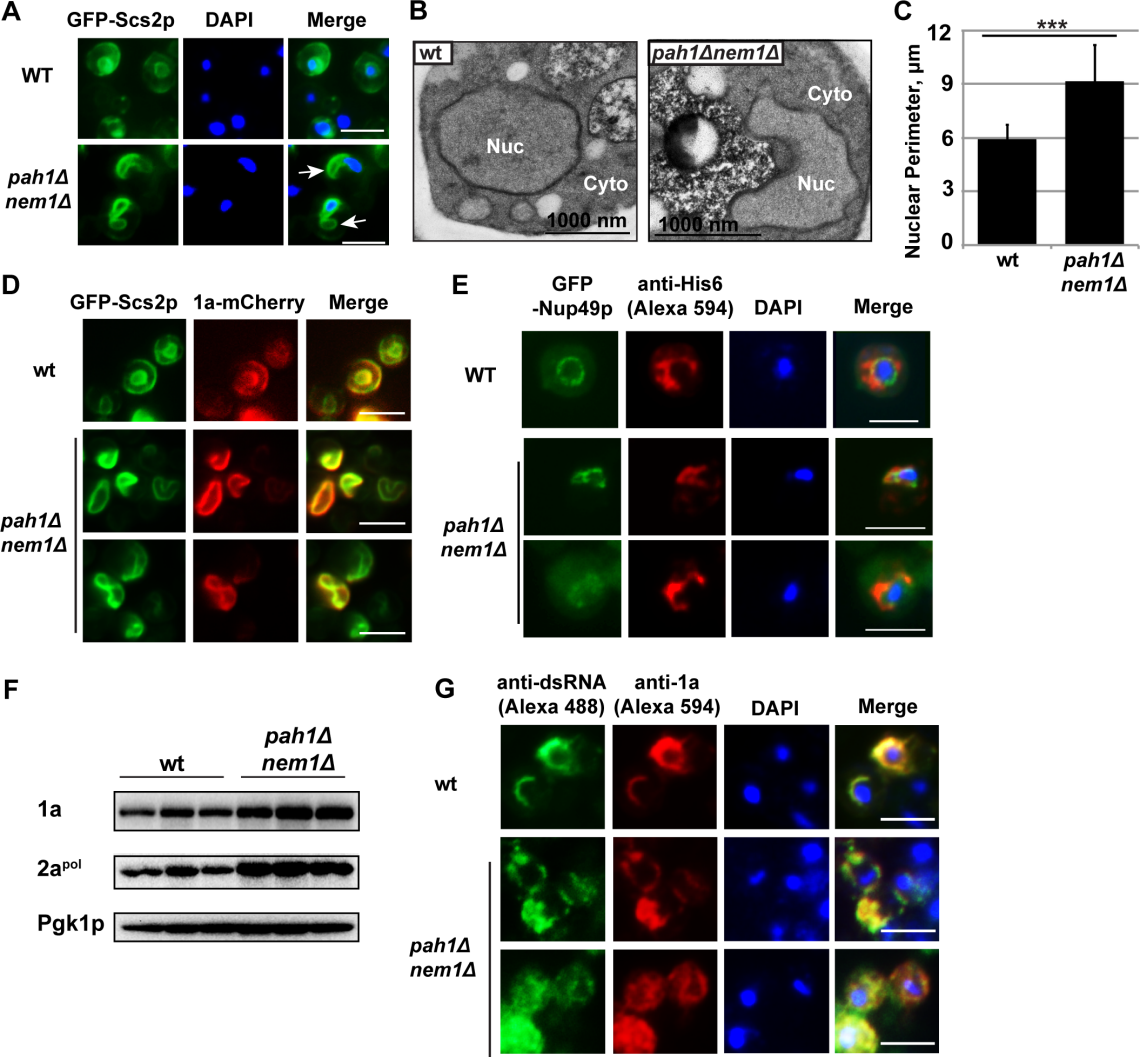
BMV 1a and double-stranded RNA localize to the extended nuclear membrane in *pah1*Δ *nem1*Δ cells. (A) Epifluorescence microscopic images of the extended nuclear membrane observed in *pah1*Δ *nem1*Δ cells. GFP tagged Scs2p, an ER membrane protein, represents ER membranes. Nuclei were stained with DAPI. White arrows indicate the extended nER, which is away from DAPI-stained chromosome DNA (blue). (Scale bar, 5μm) (B) Morphology of the nuclear membrane in wt and *pah1*Δ *nem1*Δ cells under transmission electron microscope. (C) The perimeters of nuclei in wt and *pah1*Δ *nem1*Δ cells. ***, p < 0.001 (ANOVA single factor test). (D) Epifluorescence microscopic images showing the localization of 1a in wt and *pah1*Δ *nem1*Δ cells. The localization of 1a is indicated by mCherry, which is fused to the C-terminus of 1a. The yellow color in merged images represents the co-localization of 1a and Scs2p signals. (Scale bar, 5μm) (E) Immunofluorescence microscopic images showing the localization of 1a-His6 in wt and *pah1*Δ *nem1*Δ cells. Localization of 1a-His6 was detected using a polyclonal anti-His6 antibody and followed by a secondary anti-rabbit antibody conjugated to Alexa Fluor 594. GFP tagged Nup49p, a component of nuclear pore complexes, indicates the nuclear membrane. Nuclei were stained with DAPI. (Scale bar, 5μm) (F) Accumulated BMV 1a and 2a^pol^ in wt and *pah1*Δ *nem1*Δ cells. Total proteins were extracted from the same numbers of BMV replicating-yeast cells and analyzed by Western blotting using antibodies specific to 1a and 2a^pol^. Pgk1p serves as a loading control. (G) Immunofluorescence microscopic images of the co-localization of 1a and dsRNA signals in wt and *pah1*Δ *nem1*Δ cells. BMV 1a was detected with anti-1a antiserum followed by a secondary anti-rabbit antibody conjugated to Alexa Fluor 594. dsRNA was detected by a dsRNA-specific monoclonal antibody (J2) and a secondary anti-mouse antibody conjugated to Alexa Fluor 488. The yellow color in merged images represents the co-localization of 1a and dsRNA signals. Note 1a and dsRNA appear as a half ring in wt but not in mutant cells. Nuclei were stained with DAPI. (Scale bar, 5μm).

We first examined whether the extended nER membrane was present in *pah1*Δ *nem1*Δ cells in the absence of BMV components using epifluorescence microscopy and transmission electron microscopy (TEM). ER membranes, which were identified using a GFP-tagged ER resident protein Scs2p (suppressor of choline sensitivity2, GFP-Scs2p), were observed as two-ring structures in wt cells (Fig 2A). The larger outer ring is the peripheral ER membrane, which is underneath the plasma membrane in yeast. The smaller inner ring indicates the nER membrane, which surrounds the DAPI-stained, round-shaped nucleus. Like the misshapen nER membrane in *nem1*Δ, *spo7*Δ, and *pah1*Δ mutants, the nER membrane was extended in the *pah1*Δ *nem1*Δ mutant (Fig 2A). Agreeing well with previous report, the extended nuclear membrane was away from the DAPI-stained chromosome DNA area (Fig 2A) and has been shown to be close to the nucleolus [46]. Consistent with what was observed by epifluorescence microscopy, the strikingly proliferated nER membrane was also confirmed in *pah1*Δ *nem1*Δ cells using TEM (Fig 2B). To further characterize the extended nuclear membrane in *pah1*Δ *nem1*Δ cells, we measured the perimeter of nER membranes in wt and *pah1*Δ *nem1*Δ cells. While the nuclear membrane perimeter in wt cells was ~5.9 μm, it increased to approximately 9.2 μm in the mutant, a 55% increase that was statistically significant (Fig 2C).

The expression of 1a, without other BMV components, induces spherule formation in the nER membrane of yeast [11]. We tested whether the localization of 1a could be affected in *pah1*Δ *nem1*Δ cells. To visualize 1a, we first used an mCherry-tagged 1a, which primarily localized to the nER and partially localized to the peripheral ER in wt cells (Fig 2D, upper panels) [56]. In mutant cells, 1a-mCherry dominantly co-localized with GFP-Scs2p at the extended nER membrane (Fig 2D). To further confirm that 1a was associated with the nER membrane, we used a GFP tagged nuclear pore complex component, Nup49p (Nuclear Pore 49, GFP-Nup49p) [57]. His6-tagged 1a, when expressed alone, co-localized with the GFP-Nup49p-labeled nER membrane in both wt and *pah1*Δ *nem1*Δ cells as determined by immunofluorescence microscopy (Fig 2E).

We next checked the accumulation and localization of BMV replication proteins during BMV replication. Both 1a and 2a^pol^ accumulated at higher levels in *pah1*Δ *nem1*Δ cells compared to those in wt cells based on Western blotting using anti-1a or 2a^pol^ antibodies (Fig 2F). The increased levels of 1a was consistent with the localization of 1a-mCherry and 1a-His6 along the expanded nER membrane (Fig 2D and 2E). To determine the site of BMV replication in *pah1*Δ *nem1*Δ cells, we tested the distribution of double-stranded RNA (dsRNA) using a dsRNA-specific monoclonal antibody J2. As a replication intermediate, dsRNA is considered a hallmark of viral VRCs [58] and the J2 antibody has been commonly used to confirm localization of viral replication sites [58,59]. In wt cells, dsRNA signal co-localized nicely with that of 1a, as determined by immunofluorescence microscopy (Fig 2G). Moreover, both signals showed a half-ring structure surrounding the nucleus. However, at least two alterations were noticed in the majority of *pah1*Δ *nem1*Δ cells (Fig 2G): 1) Both dsRNA and 1a signals were not detected as a half-ring but localized at the extended nER membrane, and 2) Both signals extended away from the nucleus in many cells.

### Substantially increased numbers of viral replication complexes are formed in *pah1*Δ *nem1*Δ cells compared to wild-type cells

**Fig 3:**
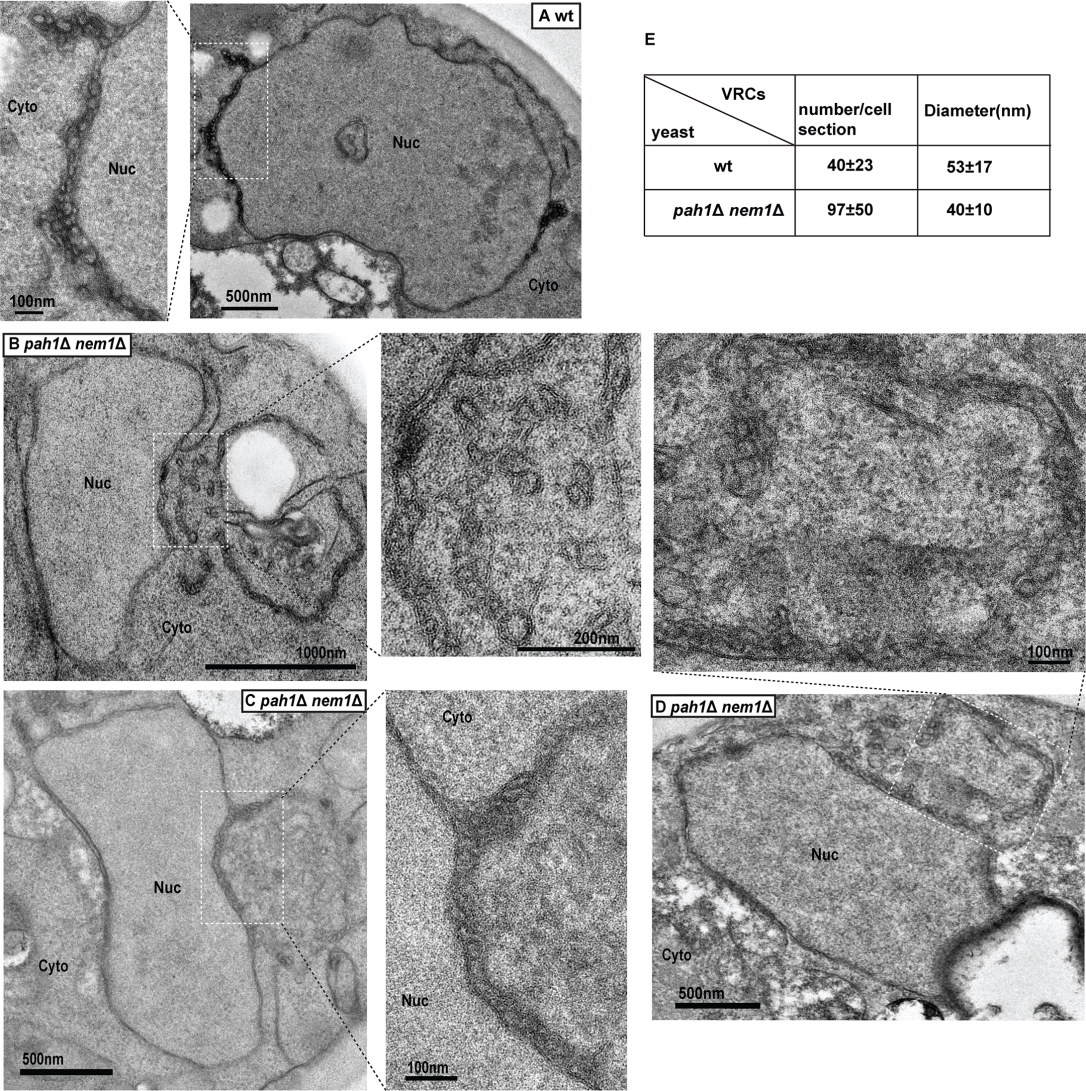
Number of spherular VRCs is substantially increased in *pah1*Δ *nem1*Δ cells. Electron micrographs of spherular VRCs formed in wt (A) and *pah1*Δ *nem1*Δ cells (B-D) are shown. Micrographs at a higher magnification of boxed areas are also shown. Note spherular VRCs are in membranes extended from the nER membrane. (E) Average number of VRCs per cell section and diameter of VRCs in wt and *pah1*Δ *nem1*Δ cells. Nuc, nucleus; Cyto, cytoplasm.

**Fig 4:**
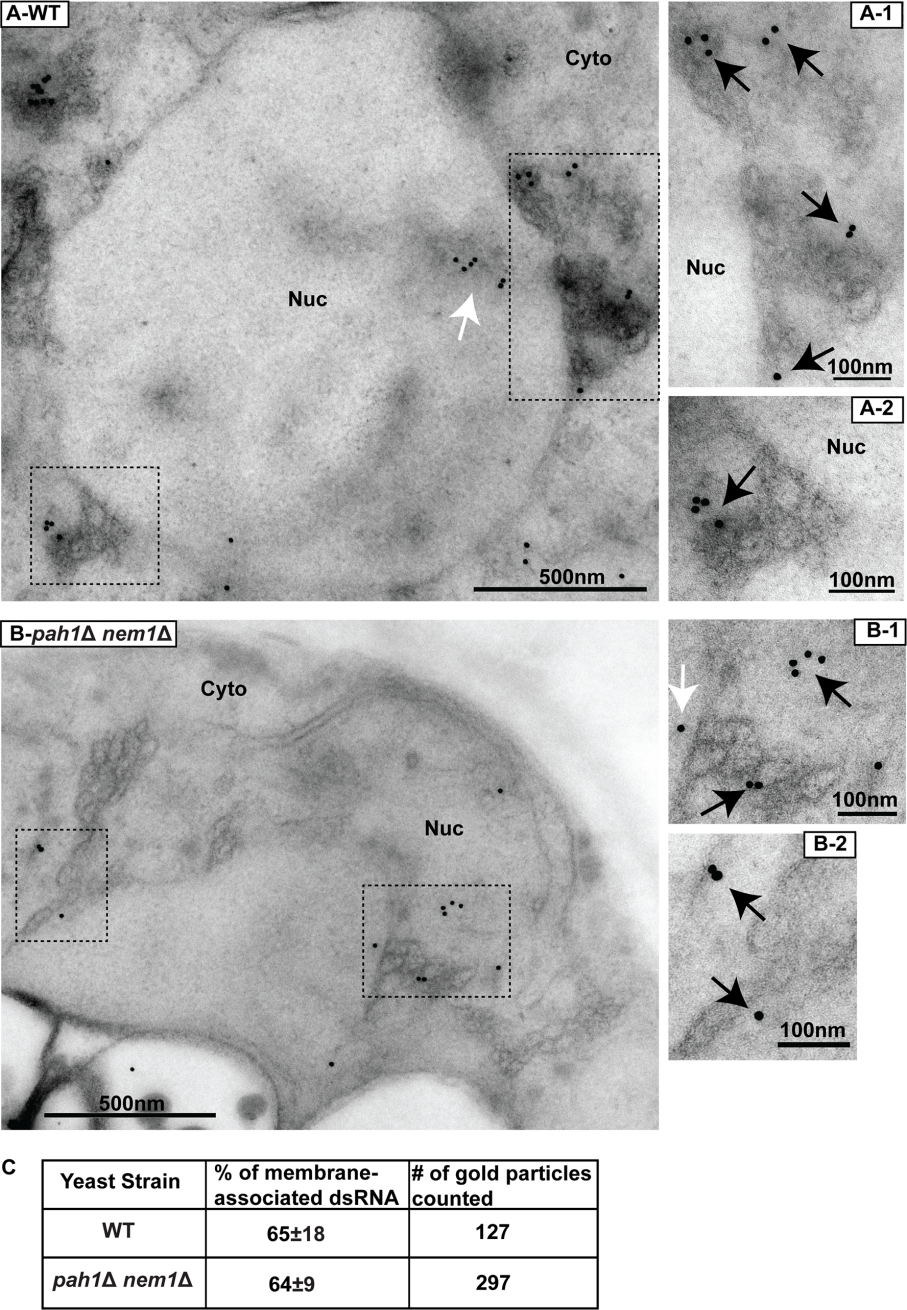
BMV replication sites are localized at the expanded nuclear ER membranes in *pah1*Δ *nem1*Δ cells. Immunogold labeling of dsRNA in wt (A) and *pah1*Δ *nem1*Δ (B) cells during BMV replication. Images at a higher magnification of the boxed areas (A-1, A-2, B-1, and B-2) are also shown. Black arrows indicate the gold particles that were associated with membranes or spherular VRCs. White arrows indicate the gold particles that were not associated with membranes. (C) Number of total particles counted and the percentage of particles that were localized to the nER membrane and spherular structures among total counted particles in wt and *pah1*Δ *nem1*Δ cells. Particles within 20 nm of the nER membrane or spherular VRCs, the distance spanned by primary and secondary antibodies, were counted as positive [98]. Nuc, nucleus; Cyto, cytoplasm.

To determine whether VRC assembly was affected in the *pah1*Δ *nem1*Δ mutant, we checked the morphology of spherular VRCs using TEM in both wt and mutant cells during BMV replication. In wt cells, viral spherular VRCs were found in the lumen of the nER membrane. In wt cells of the RS453 background, the average number of spherular VRCs per cell section was approximately 40 (40 ± 3, [mean±SD]) with an average diameter of ~53 nm (53 ± 17 nm, Fig 3A). In BMV-replicating *pah1*Δ *nem1*Δ cells, an extended nER membrane was clearly observed (Fig 3B and 3C), similar to what was seen in mutant cells without BMV components (Fig 2C). We found VRCs that were 24% smaller in diameter (40 ± 10 nm, Fig 3B–3E) but about 2.4-fold more abundant in number (97 ± 50, Fig 3B–3E) compared to those in wt cells. These spherular VRCs were generated from membranes connected to the nER membrane. The increased numbers of VRCs is consistent with higher accumulation of both BMV 1a and 2a^pol^ (Fig 2F). To confirm that these smaller VRCs were active in viral replication, we performed immunogold electron microscopy analysis (IEM) using the J2 antibody [59]. About 65% of the gold particles were associated with viral VRCs in BMV-replicating wt cells (65%±18, n = 127) (Fig 4A). A similar ratio was observed (64%±9, n = 297) in *pah1*Δ *nem1*Δ cells (Fig 4B). We have similarly detected BMV 1a in VRCs in wt and *pah1*Δ *nem1*Δ cells with similar ratios, 71% (n = 110) and 75% (n = 206), respectively (S1 Fig). Given the fact that spherular VRCs are the site of RNA synthesis and that there was an increase in the accumulation of both positive- and negative-strand RNA in the mutant cells (Fig 1B), these results suggest that the smaller spherular VRCs in mutant cells support efficient viral RNA synthesis.

In addition to smaller spherular VRCs, we also observed more dramatic membrane rearrangements in *pah1*Δ *nem1*Δ cells replicating BMV, usually multiple layers of bilayer membrane surrounding the nucleus (S2 Fig). These layers of membrane are likely generated during BMV replication because such structures have not been previously reported and were not observed in the absence of BMV replication (Fig 2B). However, the nature of and the relationship of the layers to viral replication is currently unclear and is under further investigation.

### Total phospholipid levels increase in cells lacking *PAH1* in the presence of BMV replication

**Fig 5:**
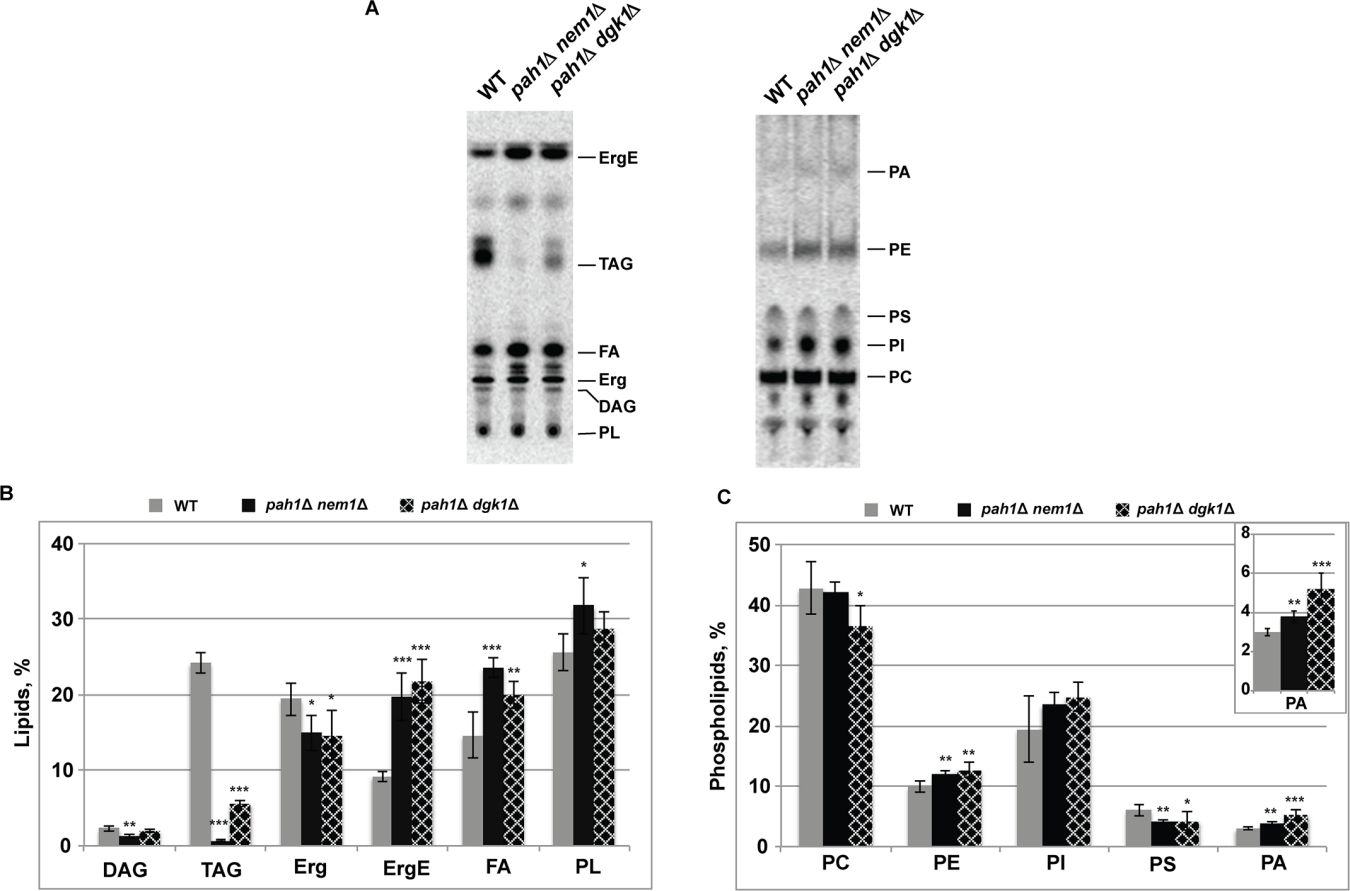
Increased total phospholipid levels in yeast cells lacking *PAH1* in the presence of BMV replication. WT, *pah1*Δ *nem1*Δ and *pah1*Δ *dgk1*Δ cells with BMV components were grown at 30°C in SC-Ura-Leu medium in the presence of galactose as the carbon source and [2-^14^C] acetate (1μCi/ml). Lipids were extracted, separated by the one-dimensional thin-layer chromatography system for phospholipids or neutral lipids, visualized by phosphoimaging and analyzed by ImageQuant software. (A) Chromatograms of neutral lipid composition and total phospholipids (*left*), and phospholipid composition (*right*). The chromatograms shown in the panel are representative of three independent experiments. (B) and (C) The mol percentages shown for the individual neutral lipids and phospholipids were normalized to the total ^14^C-labeled chloroform fraction, which also contained the unidentified neutral lipids and phospholipids shown in (A). Each data point represents the average of three experiments ± S.D.. *, P<0.05; **, P<0.01; ***, p < 0.001 (based on single factor ANOVA test).

As reported previously, levels of total phospholipids, ergosterol esters (ErgE) and free FAs increased at the expense of TAG in *pah1*Δ mutant cells [54]. To confirm that similar altered lipid composition was still present in *pah1*Δ *nem1*Δ cells in the presence of BMV replication, we measured lipids of wt and mutant cells grown in the presence of [2-^14^C] acetate to radiolabel neutral lipids and phospholipids (Fig 5A). The mol percentages of each measured lipid was reported in Fig 5B and 5C. The mol percentage of both DAG (p<0.01) and TAG (p<0.001) decreased significantly while total phospholipid levels increased (p<0.05) in *pah1*Δ *nem1*Δ mutant cells compared to those in wt cells (Fig 5B). Moreover, there was a significant decrease in ergosterol levels but a substantial increase in ErgE and free FAs levels in the presence of BMV (Fig 5B). The similar compositional changes of all aforementioned lipids, in the absence of BMV, have been previously reported [54], indicating that BMV did not alter the trend of lipid compositional changes in mutant cells.

The phospholipid composition was also altered in *pah1*Δ *nem1*Δ cells during BMV replication. Levels of PA (p<0.01) and PE (p<0.01) increased while there was a decrease in PS levels (p<0.01) in the mutant compared to wt (Fig 5C). However, there were no statistically significant changes in PC or PI levels (Fig 5C). Thus, our data agrees with a previous report [54], which showed that total phospholipid levels, PA in particular, increase upon deletion of *PAH1*, even in the presence of BMV replication.

### Extension of the nuclear membrane is not the major contributor to the increase in BMV genomic replication in cells lacking *PAH1*

**Fig 6:**
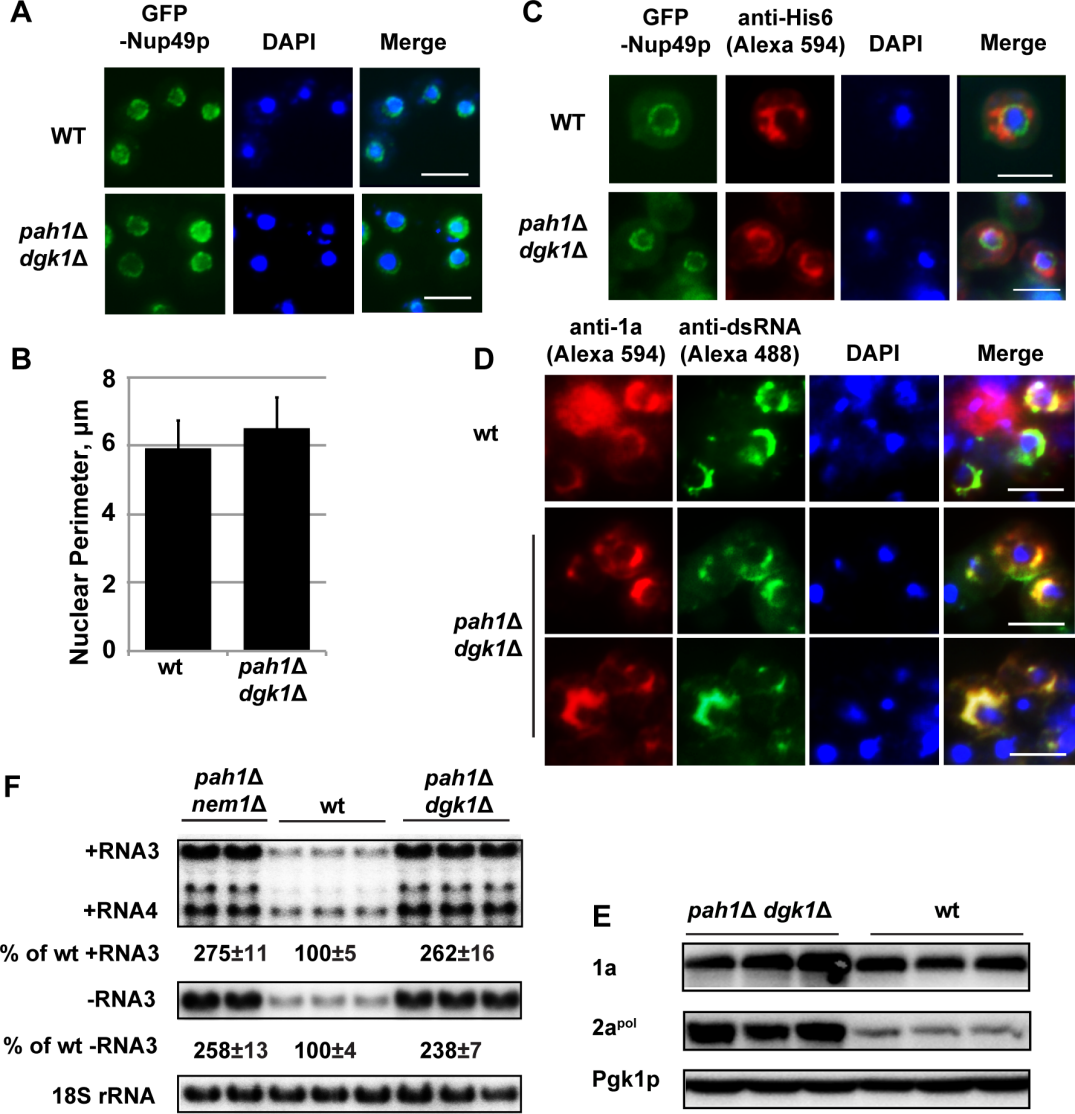
The extended nuclear membrane is not responsible for the enhanced BMV replication in cells that lack *PAH1*. (A) Epifluorescence microscopic images of the round-shaped nuclear membrane observed in wt and *pah1*Δ *dgk1*Δ cells. GFP-Nup49p was used as a nuclear membrane marker. Nuclei were stained with DAPI. (Scale bar, 5μm) (B) Nuclei perimeter measurements in wt and *pah1*Δ *dgk1*Δ cells. (C) BMV 1a localization in wt and *pah1*Δ *dgk1*Δ cells. GFP-Nup49p was used as a nuclear membrane marker. (Scale bar, 5μm) Note the wt cell (upper panel) is the same one in Fig 2E (upper panel). (D) Immunofluorescence microscopic images showing localization of dsRNA and 1a in wt and *pah1*Δ *dgk1*Δ cells. Note 1a and dsRNA appear as a half-ring structure in both wt and mutant cells. (Scale bar, 5μm) (E) Accumulated BMV 1a and 2a^pol^ in wt and *pah1*Δ *dgk1*Δ cells. Protein extraction and Western blotting were done as in Fig 2. (F) BMV replication in wt, *pah1*Δ *nem1*Δ, and *pah1*Δ *dgk1*Δ cells. Viral RNAs and 18S rRNA were detected as in Fig 1.

**Fig 7:**
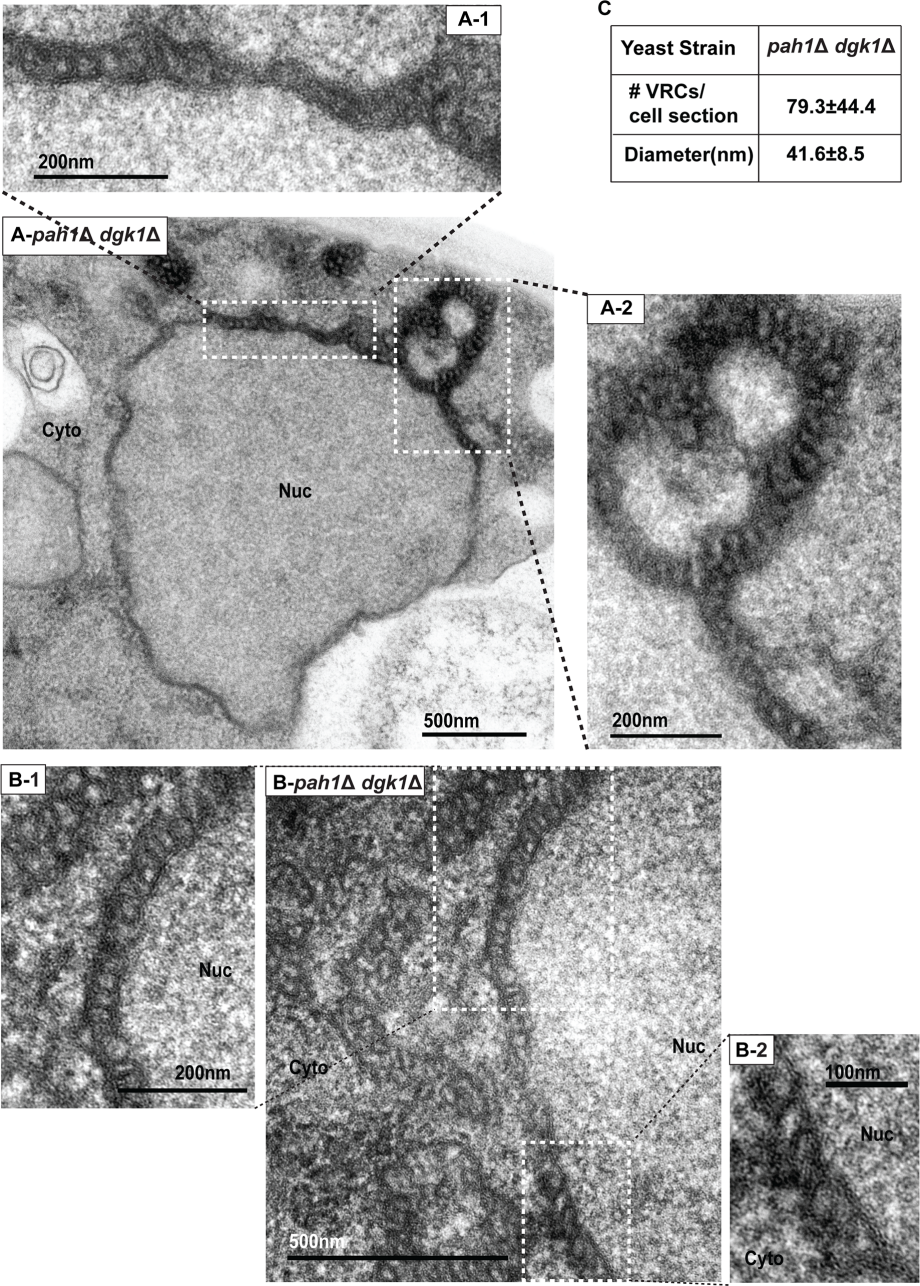
BMV replication complexes are associated with the perinuclear ER membrane in *pah1*Δ *dgk1*Δ cells. (A) and (B) Representative TEM images of spherular VRCs formed in *pah1*Δ *dgk1*Δ cells. Micrographs at a higher magnification of boxed areas (A-1, A-2, B-1, and B-2) are also shown. (C) Number and diameter of VRCs in *pah1*Δ *dgk1*Δ cells are shown. Nuc, nucleus; Cyto, cytoplasm.

In *pah1*Δ cells, several alterations may account for the enhanced BMV replication: 1) Since BMV assembles its VRCs at the nER membrane, the extended nER membrane will provide a larger surface area for the formation of BMV VRCs; 2) Since phospholipids are major components of membranes, the increased total phospholipid levels may provide building materials to form more VRCs. To determine which or both of these are the major contributor(s), we took advantage of the *pah1*Δ *dgk1*Δ mutant, in which both *PAH1* and *DGK1* are deleted. It was reported that the mutant has similar lipid compositional changes as those in the *pah1*Δ mutant but the nER membrane is normal [54]. We first checked the morphology of GFP-Nup49p-tracked nER membrane and confirmed that the nER membrane was indeed round shaped (Fig 6A) and that the size of nuclei in *pah1*Δ *dgk1*Δ cells was similar to that of wt cells (Fig 6B). The average perimeter of the nER membrane in *pah1*Δ *dgk1*Δ cells were 6.5 μm (n = 136), a 10% increase over that of wt cells at 5.9 μm (Fig 6B). However, this increase is not statistically significant. In addition, we confirmed that 1a-His6 co-localized with GFP-Nup49p in the nER membrane (Fig 6C). Consistent with the localization of 1a-His6, 1a and dsRNA were all localized at the round-shaped nER membrane during BMV replication in *pah1*Δ *dgk1*Δ cells (Fig 6D). In addition, as seen in wt cells, both 1a and dsRNA localized as a half-ring in *pah1*Δ *dgk1*Δ cells. Surprisingly, 1a and 2a^pol^ still accumulated at much higher levels compared to wt cells, even the nER membrane was not extended (Fig 6E). In addition, BMV replication increased up to ~2.5-fold, similar to that in the *pah1*Δ *nem1*Δ mutant (Fig 6F). We also observed smaller but many more abundant spherular VRCs in *pah1*Δ *dgk1*Δ cells during BMV replication compared to those in wt cells (Fig 7A and 7B). The average size of spherular VRCs was 42 ± 9 nm and the number of VRCs was about 79 ± 44 per cell section (Fig 7C). Lipid analysis indicated that the *pah1*Δ *dgk1*Δ and *pah1*Δ *nem1*Δ mutants shared similar trends in lipid compositional changes, including decreased DAG, TAG, and Erg but increased ErgE, free FAs, and total phospholipids (Fig 5B). These data indicate that the altered lipid composition, but not the extended nuclear membrane, is responsible for the enhanced BMV genomic replication in cells with disrupted Pah1p activity.

### The contribution of enhanced phospholipids in the promoted BMV replication in cells lacking *PAH1*

**Fig 8:**
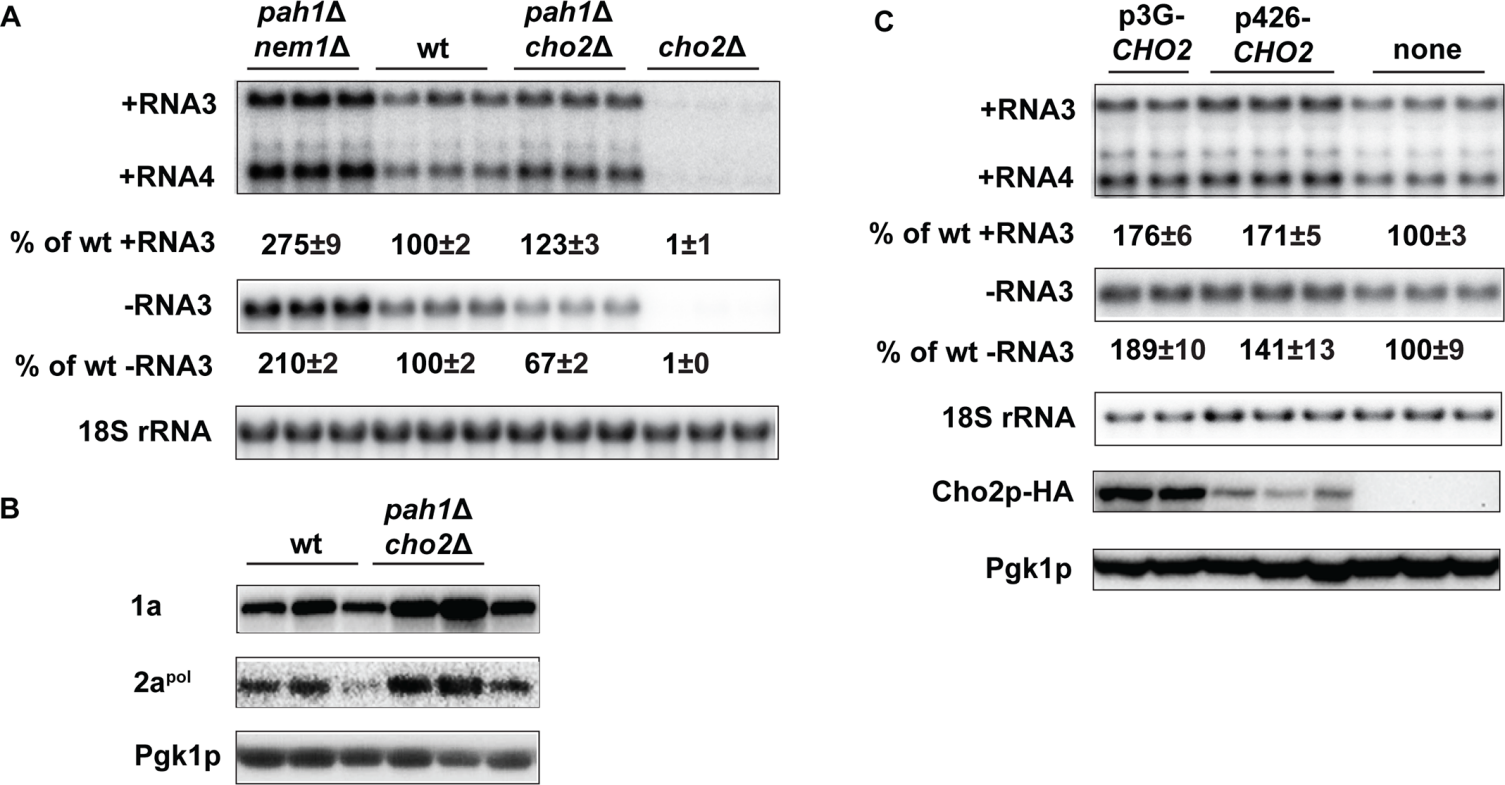
The active synthesis of phosphotidylcholine is required for the enhanced BMV replication in *pah1*Δ cells. (A) BMV replication in wt, *cho2*Δ, *pah1*Δ *cho2*Δ and *pah1*Δ *nem1*Δ cells. (B) BMV 1a and 2a^pol^ accumulation in wt and *pah1*Δ *cho2*Δ cells. Protein extraction and Western blotting were done as in Fig 2F. (C) Over-expression of *CHO2* promotes BMV replication in wt cells. Low or high levels of Cho2p-HA was expressed from p426-*CHO2* (a high-copy-number plasmid) or p3G-*CHO2* (a low-copy-number plasmid) under the control of the *CHO2* or *GAL1* promoter, respectively. The bottom panel shows accumulated Cho2p that was expressed from different vectors. Pgk1p serves as a loading control.

We further tested whether increased total phospholipid levels, among lipid compositional changes, could play an important role in promoting VRC formation and BMV replication because phospholipids are major membrane components and both ErgE and free FAs are not present in membranes. We have previously shown that a pool of PC is synthesized in the site of viral replication by recruiting host Cho2p [27], which is involved in converting PE to PC (Fig 1A) [60]. We first deleted *CHO2* and found that BMV replication was hardly detectable in the *cho2*Δ mutant in the RS453 background (Fig 8A). When *CHO2* and *PAH1* were simultaneously deleted, positive-strand RNA3 accumulation increased by 23% but negative-strand RNA3 levels decreased by 33% compared to those in wt cells. However, comparing to that in the *pah1*Δ *nem1*Δ mutant, BMV replication significantly reduced (Fig 8A). Of note, 1a and 2a^pol^ proteins increased in the *pah1*Δ *cho2*Δ mutant background compared to those in wt cells (Fig 8B).

As a result of the increase in PA levels in the *pah1*Δ mutant, transcription of phospholipid synthesis genes increases due to the sequestration of the transcription repressor Opi1p (Fig 1A) [36]. To simulate those conditions, we tested whether enhanced *CHO2* expression would promote BMV replication. To achieve different levels of overexpression in wt cells, *CHO2* was expressed from a high-copy-number plasmid under its endogenous promoter (p426-*CHO2*) or from the strong *GAL1* promoter (p3G-*CHO2*), respectively (Fig 8C). An increase of 40% or 90% of negative-strand RNA3 over that in wt cells was associated with different levels of overexpressed Cho2p (Fig 8C). An approximate 70% increase in positive-strand RNA was also noticed when *CHO2* was overexpressed (Fig 8C). However, these increases in positive- and negative-strand RNA synthesis was not as significant as that in *pah1*Δ cells, suggesting other phospholipids besides PC contribute to the enhanced BMV replication phenotype in *pah1*Δ cells (Fig 1B).

### Deleting *PAH1* improves yeast cell growth during BMV replication

**Fig 9:**
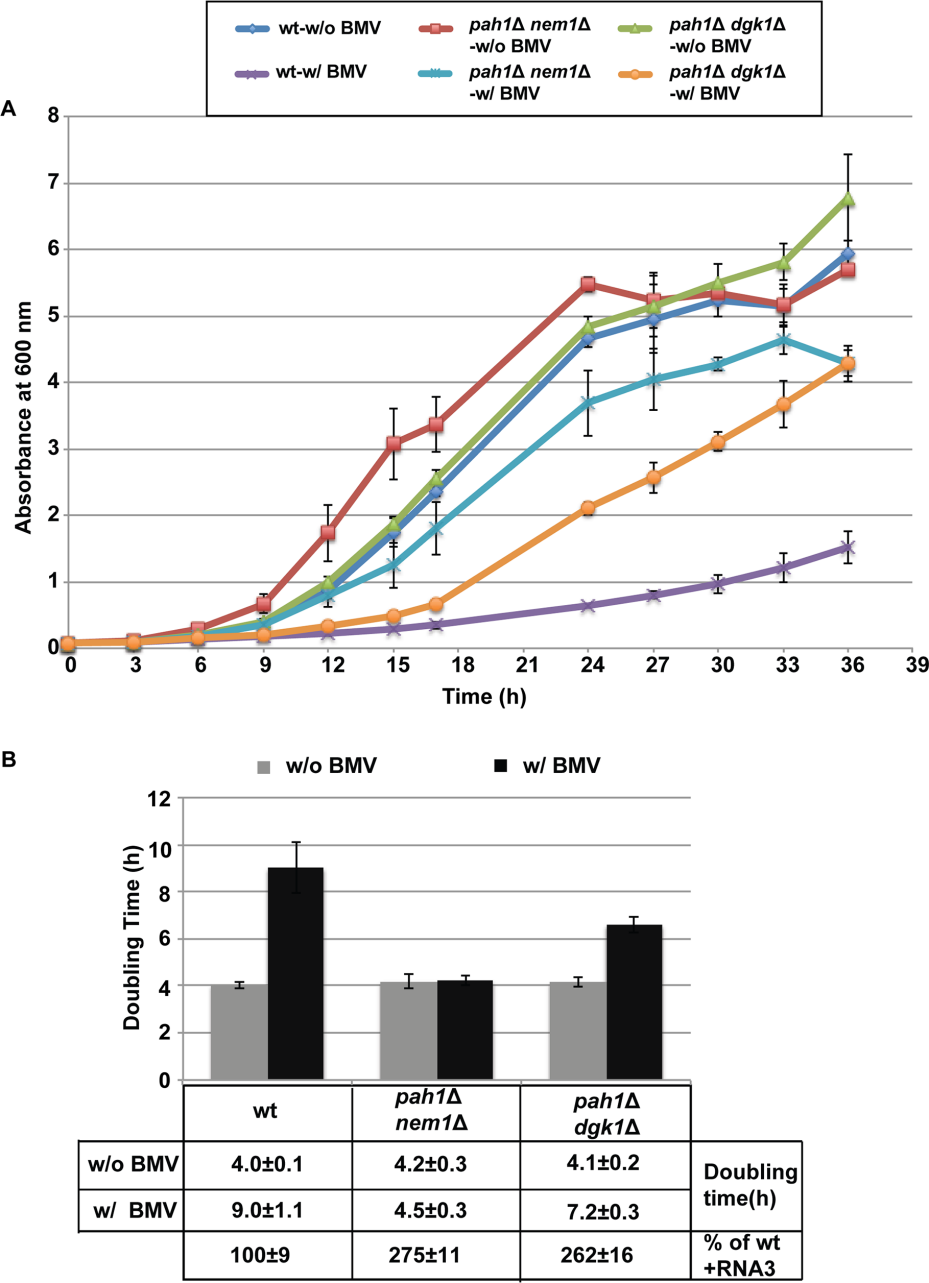
Deleting *PAH1* alleviates BMV-repressed host cell growth. Cells of wt or mutants were grown in media using galactose as the carbon source in the absence or presence of BMV replication. (A) Growth curves of wt, *pah1*Δ *nem1*Δ, and *pah1*Δ *dgk1*Δ cells in the absence or presence of BMV replication in 36 hours. (B) Doubling time of yeast strains in the absence or presence of BMV replication during exponential phase. Doubling time was calculated using the following equation: Doubling time = [hours cells grown * Ln(2)]/[Ln(A_600 nm_ at the end / A_600 nm_ at the start)].

Phospholipids are the major components of cellular membranes [60] and are utilized by various viruses for infection [5,27,61–63]. Viruses compete with their hosts for limited resources and, as a direct result, viral infections usually affect cell growth. We measured cell growth and calculated doubling times (based on growth during the exponential stage) of wt, *pah1*Δ *nem1*Δ, and *pah1*Δ *dgk1*Δ cells in the absence or presence of BMV in the galactose medium, which is to induce BMV replication (Fig 9A). BMV replication substantially slowed down the growth of wt cells. In wt cells, the doubling time increased from ~4 hours/generation in the absence of BMV to ~9 hours/generation in the presence of BMV, an approximately 2-fold increase (Fig 9B). In addition, the cell density of the culture expressing BMV components never reached to that of cells without BMV. Deleting *PAH1* profoundly improved the growth of cells with BMV replication (Fig 9). The doubling times of *pah1*Δ *nem1*Δ and *pah1*Δ *dgk1*Δ mutants in the presence of BMV replication were approximately 4.5 and 7.2 hours/generation, respectively. It should be noted that these mutant cells grew at the same rate as wt cells in the absence of BMV components (Fig 9), indicating that the growth differences between wt cells and the above mutants are directly related to BMV replication.

### Expression of plant *PAH1* orthologs inhibits BMV genomic replication in yeast and *Nicotiana benthamiana* plants

**Fig 10:**
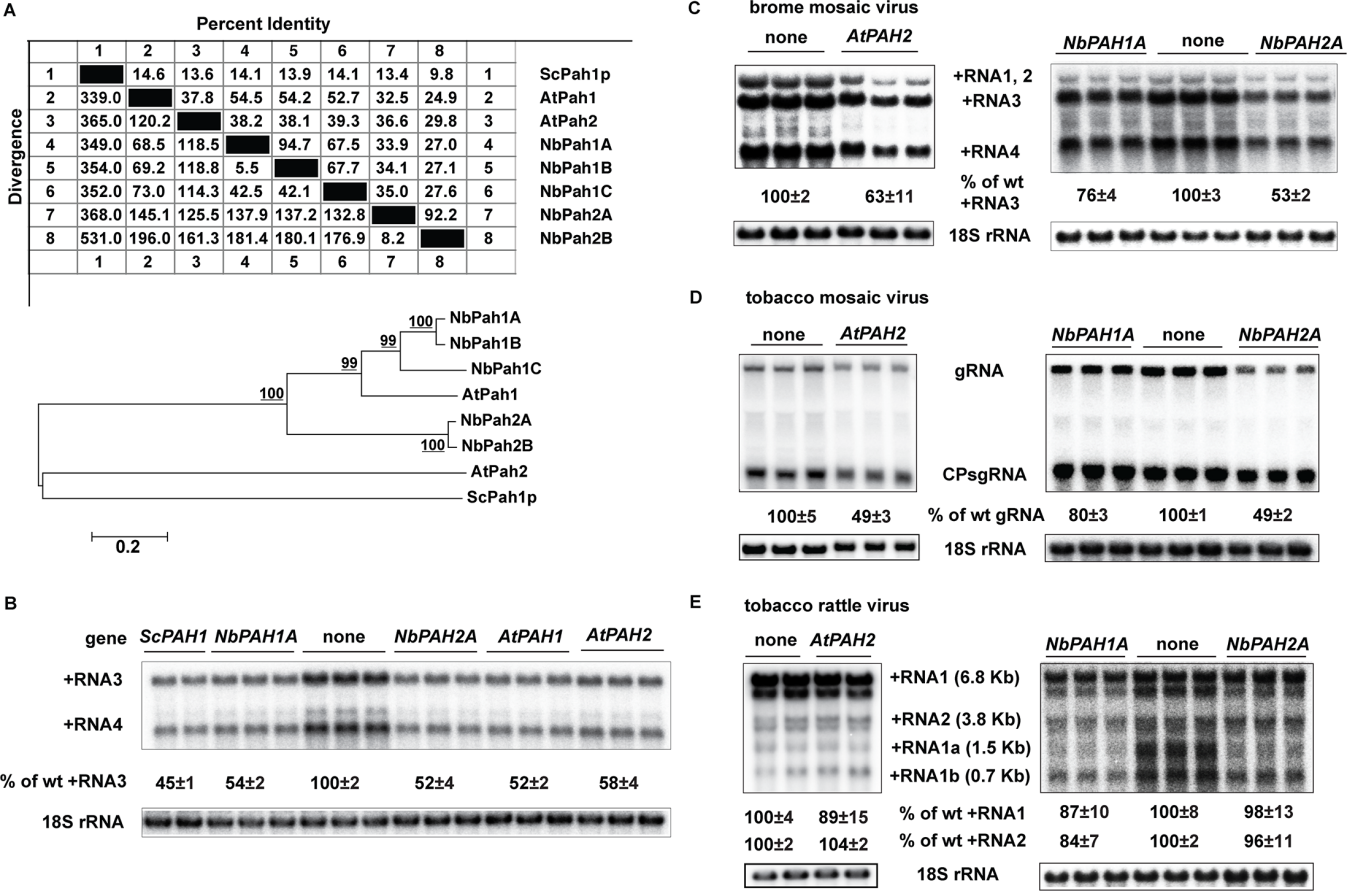
Expression of plant *PAH1* homologs inhibits BMV replication in yeast and *Nicotiana benthamiana*. (A) Homology analysis and phylogenetic tree of Pah1 proteins from yeast (ScPah1p), Arabidopsis (AtPah1 and AtPah2), and *N*. *benthamiana* (NbPah1A, 1B, 1C, 2A, and 2B). (B) BMV replication in wt yeast cells expressing *ScPAH1*, *AtPAH1*, *AtPAH2*, *NbPAH1A* or *NbPAH2A*. Positive-strand viral RNAs as well as 18S rRNA were detected as in Fig 1. Genome replications of BMV (C), TMV (D), or TRV (E) in *N*. *benthamiana* leaves expressing *AtPAH2*, *NbPAH1A* or *NbPAH2A*. BMV, TMV, and TRV were launched by agroinfiltration in *N*. *benthamiana* leaves 2 days after agroinfiltration to express the plant *PAH1* orthologs. Virus-infected leaves were harvested 3 days after agroinfiltration. RNA was extracted and viral positive-strand RNA was detected by using virus strand-specific probes as in Fig 1.

The PAP enzyme is present in yeast, plants and humans [64]. The two *PAH1* orthologs in *Arabidopsis thaliana* (*AtPAH1* and *AtPAH2*) could complement the phenotypical defects in *pah1*Δ cells [47–49] even though *Arabidopsis* and yeast Pah proteins share only ~14% identity at the protein level (Fig 10A). We have additionally identified five putative *PAH* genes in the genome of *Nicotiana benthamiana* based on the sequence similarity to *Arabidopsis AtPAH1* and *AtPAH2*: *NbPAH1A* (Niben101Scf01009g01015.1), *NbPAH1B* (Niben101Scf05306g01007.1), *NbPAH1C* (Niben101Scf07223g03002.1), *NbPAH2A* (Niben101Scf05628g01019.1), and *NbPAH 2B* (Niben101Scf08200g05005.1). They can be classified into two clades, *NbPAH1A*, *1B*, and *1C* as one clade and *NbPAH2A* and *2B* as the other one, based on their sequence similarity to *AtPAH1* and *AtPAH2* and among themselves (Fig 10A). To test the role of plant *PAHs* in BMV genomic replication, we expressed *NbPAH1A*, *NbPAH2A*, *AtPAH1* or *AtPAH2* in yeast cells to test whether their expression could inhibit BMV replication in a similar manner to that of yeast *PAH1* (Fig 1D). All genes were expressed from a high-copy-number plasmid under the control of the *GAL1* promoter [49]. Like yeast *PAH1*, the expression of plant orthologs inhibited BMV replication by ~40–50% (Fig 10B).

We next tested how BMV genome replication was affected when plant *PAH1* orthologs were highly expressed in *N*. *benthamiana*, which is a systemic host for BMV [13,65,66] and serves as a universal host for plant viruses [67]. *AtPAH2*, *NbPAH1A* and *NbPAH2A* were expressed from an enhanced cauliflower mosaic virus (CaMV) 35S promoter by agroinfiltration. BMV genome replication was inhibited by 40–50% based on the accumulation of positive-strand RNA3 when *AtPAH2* or *NbPAH2A* was expressed. However, the expression of *NbPAH1A* only inhibited BMV replication by ~25%, suggesting that different plant *PAH1* orthologs may play different roles in plants (Fig 10C).

To determine whether the inhibition of viral replication by plant *PAH1* orthologs was specific to replication of BMV or if it was a general effect on other plant (+)RNA viruses, we also tested tobacco mosaic virus (TMV) and tobacco rattle virus (TRV). TMV replicates in association with ER membranes while TRV replicates on mitochondrial membranes [68,69]. We previously included both viruses and showed that a dominant negative mutant of *AtSNF7-2* (sucrose nonfermenting7) did not affect the replication of TMV and TRV but specifically affected BMV [70]. Here we found that TMV genome replication was inhibited by ~50% when *AtPAH2* or *NbPAH2A* was expressed in *N*. *benthamiana*. However, TMV replication was only slightly inhibited by the *NbPAH1A* expression (Fig 10D), which similarly inhibited BMV replication at a lesser degree compared to *AtPAH2* and *NbPAH2A* (Fig 10C). On the contrary, based on two genomic RNAs, RNA1 and 2, TRV genome replication was not significantly affected by any of plant orthologs in *N*. *benthamiana* (Fig 10E). However, the accumulation of its subgenomic RNA1a levels were lower than that in untreated plants (Fig 10E). It is unclear why the accumulation of different TRV RNAs was differently affected by the overexpression of *NbPAHs*.

## Discussion

We report here that the host enzyme phosphatidic acid phosphohydrolase restricts BMV RNA replication by limiting the phospholipid synthesis and that BMV takes advantage of altered lipid composition, including the increased total phospholipids, in yeast cells lacking *PAH1* to assemble many more VRCs and substantially promote its genomic replication. Although deleting *PAH1* leads to several phenotypes that could facilitate BMV replication, our data suggest that the increased levels of total phospholipids but not the proliferated nER membrane is the primary contributor (Figs 5 and 6). The rise in levels of total phospholipids in cells lacking *PAH1*, possibly with other altered lipids, also significantly improved cell growth during viral replication. It has been reported that in *pah1*Δ cells TBSV assembles its VRCs and replicates robustly in the extended ER membranes [52], but the improved replicase activity is primarily responsible for the enhanced TBSV replication [51]. Thus, our work complements and expands the current understanding of Pah1p’s role in balancing cellular phospholipid and storage lipid synthesis as well as in replication of various viruses.

### Enhanced levels of total phospholipids, not the extended nuclear membrane, are primarily responsible for the increased BMV replication in cells that lack *PAH1*

Although both BMV and TBSV replicate at much higher levels in *pah1*Δ cells compared to wt cells, the mechanisms by which each virus takes advantage of *PAH1* deletion to improve its replication are different. Under normal conditions TBSV forms spherular VRCs in peroxisomes, however, it preferentially assembles VRCs in association with extended ER membranes in *pah1*Δ *nem1*Δ cells [51,52]. It is not specified whether more VRCs are formed in *pah1*Δ *nem1*Δ cells [52], but it is clear that TBSV VRCs isolated from the mutant cells are more efficient in supporting viral RNA synthesis *in vitro* than those from wt cells [51]. Our TEM data showed that in *pah1*Δ *nem1*Δ cells, viral spherules were present in the extended nER membrane and were about 2.4-fold more abundant in number than those in wt cells (Fig 3B–3D). Despite the fact that the nuclear membrane was normal in *pah1*Δ *dgk1*Δ cells (Figs 6 and 7) [54], BMV replication levels and the number of VRCs were similar in the *pah1*Δ *dgk1*Δ and *pah1*Δ *nem1*Δ mutants (Figs 6F and 7), indicating that the expanded nER membrane is not the major factor in promoting the VRC formation and viral genomic replication.

Two lines of evidence support that increased total phospholipid levels played an important role in the enhanced BMV replication in *pah1*Δ cells: (1) Reducing PC synthesis by deleting *CHO2* in the *pah1*Δ mutant diminished the substantially enhanced BMV replication (Fig 8A); (2) Overexpressing *CHO2* in wt cells enhanced BMV replication (Fig 8C). However, the enhancement in BMV genomic replication was not as significant as that in *pah1*Δ cells because total phospholipids, not just PC, increased in *pah1*Δ cells.

There are increases in three major lipids in cells lacking *PAH1*, either in the absence [54] or presence of BMV (Fig 5B), total phospholipids, ErgE, and free FAs. We focused on the roles of increased total phospholipids in this work because ErgE is not present in membranes and its involvement in genomic replication of any viruses has not been reported. Although it was reported previously that the expression of BMV 1a enhanced the accumulation of total FAs by 33% per yeast cell [24], it should point out that what we measured here was free FAs, which were not incorporated into phospholipids and not present in membranes. How are free FAs involved in BMV replication is not clear. Nevertheless, it merits further investigation whether ErgE or free FAs is involved in BMV replication. Although phospholipids are major building blocks of membranes and our work suggested that increased total phospholipids play an important role in the enhanced BMV replication (Fig 8), the possible contribution of other significantly altered lipids, such as ErgE and free FAs, cannot be totally ruled out.

### Deleting *PAH1* facilitates both BMV replication and host cell growth

Lipid-containing cellular membranes are the sites where (+)RNA virus replication invariably takes place, although different viruses exploit specific organelle membranes and require different lipid microenvironment for their replication [1,4–6]. For example, TBSV requires a PE-enriched microenvironment [61,71]. Phosphatidylinostol-4-phosphate (PI4P) is produced in VRCs by hepatitis C virus (HCV)-recruited phosphatidylinositol-4-kinase III α (PI4KIIIα) [72,73] or Coxsackievirus B3-engaged PI4KIIIβ [74] either for the assembly or proper function of the VRCs, respectively. Our prior work showed that PC content is enriched at the viral replication sites of a number of (+)RNA viruses, including BMV, HCV, and poliovirus [27].

It has recently been reported that *pah1*Δ cells are susceptible to abiotic stresses and have a short chronological life span [75]. Our data showed that deleting *PAH1* substantially improved host cell growth during viral replication. Yeast mutants that lack *PAH1*, either by itself or in combination with a second mutation, divided at a faster rate than wt cells during BMV replication (Fig 9). It should be noted that the mutant cells divided at a similar rate to wt cells in the absence of BMV replication (Fig 9). Both *pah1*Δ *nem1*Δ and *pah1*Δ *dgk1*Δ mutants grew faster than wt, suggesting that both of the increased viral replication and host cell growth were likely due to changes in lipid composition, possibly to the increase in total phospholipid levels. These results are consistent with the notion that BMV competes for the limited intracellular phospholipid resources with host cells, and that increasing total phospholipid levels could satisfy the requirements for both viral replication and host cell growth. As such, Pah1p serves as a limiting factor for BMV and possibly other (+)RNA viruses by directing lipid synthesis away from phospholipid synthesis, via converting PA to storage lipids. It is also possible that Pah1p promotes storage lipid synthesis at the onset of viral replication as a host reaction to stresses imposed by viral infection and in turn, limits cell growth.

### Possible roles of PA in regulating nuclear membrane morphology and viral replication

In eukaryotic cells, besides serving as a key intermediate in lipid synthesis, PA is involved in multiple biological processes as a signaling molecule, such as cell growth and proliferation, secretion, endocytosis, and vesicular trafficking in mammalian cells [76–79] as well as responses to biotic and abiotic stress and seed germination in plants [78–83].

Increased PA may be involved in viral replication through several nonexclusive mechanisms. One is through the extension of the nER membrane, which could provide more room for VRC assembly, such as the substantially enhanced replication of TBSV and related viruses [51]. Another possible mechanism is that higher PA levels may recruit PA-dependent effectors. As a signaling lipid, PA executes its function by binding to effector proteins and recruiting them to a specific membrane [79]. Because such binding is dependent on the concentration of PA in the bilayer, higher levels of PA in the nER membrane may recruit its effectors more efficiently [79]. Some of these effectors may play positive, yet unclear, roles in (+)RNA virus replication. This is supported by the enhanced BMV replication in the *pah1*Δ *dgk1*Δ mutant (Fig 6), which has wt nER membrane but enhanced levels of total phospholipids including a high level of PA [54]. A third possible option is that the enhanced accumulation of PA and other phospholipids may affect protein conformation and stability. This is supported by increased accumulations of 1a and 2a^pol^ in both *pah1*Δ *nem1*Δ and *pah1*Δ *dgk1*Δ mutants (Figs 2F and 6E). It is also possible that PA may play a direct role in the formation of VRCs because the incorporation of PA, a cone-shaped lipid, promotes the formation of negative curvature [84–86]. BMV spherules are formed by invaginating the outer nER membrane away from the cytoplasm, thus inducing a negative curvature. Higher PA levels may facilitate the formation of viral spherules, which may explain significantly increased numbers of viral VRCs formed in *pah1*Δ *nem1*Δ (Fig 3) and *pah1*Δ *dgk1*Δ cells (Fig 7).

Besides *de novo* synthesis, PA can be produced by phospholipase D (PLD)-catalyzed removal of the choline head group from PC. PLD-generated PA plays an important role in supporting replication of plant (+)RNA viruses [87]. The replication protein p27 of red clover necrotic mosaic virus (RCNMV) in the *Tombusviridae* family binds to PA directly. Knocking down the expression or inactivation of PLD severely inhibited RCNMV replication [87]. It should be noted that inhibition of PLD activity by addition of *n*-butanol in tobacco protoplasts also inhibited BMV replication, indicating an important role of PA in the replication of a group of plant (+)RNA viruses [87]. Our data in yeast agree with the important role of PA in BMV replication in plants, although the sources of the increased PA are different.

In summary, our work suggests that altered lipid composition, likely through the enhanced total phospholipids, is the major factor not only for promoting BMV genomic replication but also for alleviating the virus-repressed cell growth in cells lacking Pah1p. Our data complement and extend prior findings on the role of PA in lipid metabolism and virus infections.

## Materials and methods

### Yeast strains and growth condition

All yeast strains used in this study are listed in Table 1 and were derived from the strain RS453 (*MATa ade2-1*, *his3-11*, *15 leu2-3*, *ura3-52*, *112 trp1-1*). The *spo7*Δ mutant was generated by replacing *SPO7* with a *HIS3MAX6* cassette. The *pah1*Δ *nem1*Δ mutant was made by replacing *NEM1* with a *KanMAX4* cassette in the *pah1*::*TRP1* background. In the majority of experiments presented, the *pah1*Δ *nem1*Δ mutant was used.

**Table 1:** Yeast strains used in this study.

Strain	Genotype	Ref./source
**RS453**	*MATa ade2-1*, *his3-11*, *ura3-52*, *15 leu2-3*, *112 trp1-1*	[54]
***nem1***Δ	RS453 *nem1*::*HIS3*	[53]
***pah1***Δ	RS453 *pah1*::*TRP1*	[54]
***spo7***Δ	RS453 *spo7*::*HIS3*	This study
***cho2***Δ	RS453 *pah1*::*TRP1 cho2*::*KanMX4+*YCplac33-URA3-*PAH1*	[54]
***nem1***Δ ***spo7***Δ	RS453 *nem1*::*HIS3 spo7*::*HIS3*	[53]
***pah1***Δ ***cho2***Δ	RS453 *pah1*::*TRP1 cho2*::*KanMX4*	[54]
***pah1***Δ ***nem1***Δ	RS453 *pah1*::*TRP1 nem1*::*KanMX4*	This study
***pah1***Δ ***dgk1***Δ	RS453 *pah1*::*TRP1 dgk1*::*HIS3*	[54]

Yeast cells were grown at 30°C in synthetic complete (SC) medium containing 2% galactose as the carbon source. Histidine, leucine, uracil, or combinations of them were omitted from the medium depending on the selection markers of plasmids [26]. After two passages (24~48 hours) in SC medium, cells were harvested when the absorbance at 600 nm (A_600 nm_) reached between 0.4–1.0 [26].

### Plasmids and antibodies

The plasmids used in this study are shown in Table 2. To launch BMV replication in yeast, plasmids pB12VG1 and pB3VG128 were used in the experiments as described before [26]. In the pB12VG1 plasmid, 1a is controlled by the *GAL1* promoter while 2a^pol^ is under the control of the *GAL10* promoter. RNA3 is under the control of the copper-inducible *CUP1* promoter but no copper was purposely included in the medium. *DGK1*, *DGK1*-*D177A* are overexpressed from a low-copy number plasmid YCplac33 under the control of the *GAL1* promoter and tagged with HA. The plasmid pB1YT3-mCherry was used to express mCherry-tagged BMV 1a. Rabbit anti-1a antiserum (a gift from Dr. Paul Ahlquist at University of Wisconsin-Madison), mouse anti-His6 (Genescript, 6G2A9), mouse anti-dsRNA J2 antibody (English and Scientific Consulting, Hungary), and rabbit anti-HA (Thermo Fisher Scientific, 71–5500) were used at 1:100 dilution for Immunofluorescence microscopy and 1:10,000 or 1:3,000 for Western blotting. For Western blotting, we also used mouse anti-BMV 2a^pol^ at 1:3,000 dilution, and mouse anti-Pgk1p (Invitrogen, 459250) at 1:10,000 dilution.

**Table 2:** Plasmids used in this study.

Plasmid	Description	Ref./source
**pB12VG1**	BMV 1a and 2a^pol^ are driven by *GAL1* or *GAL10* promoter respectively in a CEN/LEU vector	[27]
**pB3VG128-U**	BMV RNA3 is under control of *CUP1* promoter in a CEN/URA vector	[27]
**pB3VG128-H**	BMV RNA3 is under control of *CUP1* promoter in a CEN/HIS vector	[27]
**pB1YT3**	BMV 1a is under control of *GAL1* promoter in a CEN/URA vector	[27]
**pB1YT3-mCherry-L**	1a-mCherry is under control of *GAL1* promoter in a CEN/LEU vector	[56]
**pUN100-GFP-*NUP49***	GFP-Nup49p is constructed in pUN100, a CEN/LEU vector	[88]
**p3G-*DGK1*-HA**	*DGK1* is under control of *GAL1* promoter in a CEN/URA vector	This study
**p3G-*DGK1*-*D177A*-HA**	*dgk1-D177A* is under control of *GAL1* promoter in a CEN/URA vector	This study
**pBG1805-*SPO7***	*SPO7* is under control of *GAL1* promoter in the pBG1805, 2μ/URA vector	[89]
**pBG1805-*NEM1***	*NEM1* is under control of *GAL1* promoter in the pBG1805, 2μ/URA vector	[89]
**pBG1805-*PAH1***	*PAH1* is under the control of *GAL1* promoter in the pBG1805, 2μ/URA vector	[89]
**pBG1805-*SKI8***	*SKI8* is under control of *GAL1* promoter in the pBG1805, 2μ/URA vector	[89]
**p3G-*CHO2*-HA**	*CHO2* is under control of *GAL1* promoter in a CEN/URA vector	[27]
**p426-*CHO2*-HA**	*CHO2* is under control of *CHO2* endogenous promoter in a 2μ/URA vector	This study
**YCplac33-*PAH1***	*PAH1* is under control of its endogenous promoter in a CEN/URA vector	[54]
**p3G-*PAH1*-HA**	*PAH1* is under control of *GAL1* promoter in a CEN/URA vector	This study
**pYes2-*AtPAH1***	*AtPAH1* is under control of *GAL1* promoter in the pYES2.1/NT 2μ/URA vector	[49]
**pYes2-*AtPAH2***	*AtPAH2* is under control of *GAL1* promoter in the pYES2.1/NT 2μ/URA vector	[49]
**pYes2-*NbPAH1A***	*NbPAH1A* is under control of *GAL1* promoter in the pYES2.1/NT 2μ/URA vector	This study
**pYes2-*NbPAH2A***	*NbPAH2A* is under control of *GAL1* promoter in the pYES2.1/NT 2μ/URA vector	This study
**pPWHT- *NbPAH1A***	*NbPAH1A* is under control of an enhanced CaMV 35S promoter	This study
**pPWHT- *NbPAH2A***	*NbPAH2A* is under control of an enhanced CaMV 35S promoter	This study
**pAG2p-*AtPAH2***	*AtPAH2* is under control of an enhanced CaMV 35S promoter	This study

### RNA extraction and Northern blotting

Total RNA was extracted using a hot phenol method [90]. Equal amounts of total RNA were used for Northern blotting analysis. P^32^-labled probes specific to BMV positive- or negative-strand RNA or 18S rRNA were used in the hybridization. Radioactive signals were scanned using a Typhoon FLA 7000 phosphoimager and the intensity of radioactive signals were quantified by using ImageQuant TL (GE healthcare). The 18S rRNA signal was used to normalize BMV RNA signals to eliminate loading variations [26].

### Western blotting

Two A_600 nm_ units of yeast cells were harvested and total proteins were extracted as described previously [25]. Equal volumes of total proteins were separated by 10% sodium dodecyl sulfate polyacrylamide gel electrophoresis (SDS-PAGE) and transferred to polyvinylidene difluoride (PVDF) membrane. Rabbit anti-BMV 1a (1:10,000 dilution), mouse anti-BMV 2a^pol^ (1:3,000 dilution), rabbit anti-HA (1:5,000 dilution), and mouse anti-Pgk1p (1:10,000 dilution) were used to detect 1a, 2a^pol^, HA, and Pgk1p [26]. Pgk1p was used as a loading control. Horseradish peroxidase (HRP)-conjugated anti-rabbit or anti-mouse antibodies (Thermo Fisher Scientific 32460 or 32430, 1:5,000 dilution) together with Supersignal West Femto substrate (Thermo Fisher Scientific, 34096) were used for signal detection.

### Electron microscopy

Samples were prepared as described previously [26]. Briefly, 10 A_600 nm_ units of cells were fixed with 4% paraformaldehyde and 2% glutaraldehyde for 1 hour followed by secondary fixation in 1% osmium tetroxide for another 1 hour. After dehydration through an ethanol gradient, yeast cells were embedded in Spurr’s resin (Electron Microscopy Sciences) for overnight. The sample sections were stained with uranyl acetate and lead citrate and observed under a JEOL JEM 1400 TEM at the Virginia-Maryland College of Veterinary Medicine.

For immunogold labeling, 4% paraformaldehyde and 0.5% glutaraldehyde were used to fix 10 A_600 nm_ units of cells for 1 hour and followed by 0.1% osmium tetroxide secondary fixation for another 15 minutes. After dehydration through an ethanol gradient, yeast cells were embedded in LR White resin (Electron Microscopy Sciences) for overnight. Embedded samples were sectioned and nickel grids were used to hold the samples. After treated with blocking solution (AURION) for 30 minutes, grids were incubated with primary antibody diluted in incubation buffer (PBS, pH7.4, 0.15% AURION BSA-c and 15mM NaN_3_) and secondary antibody conjugated with colloidal gold particles (10nm or 15nm particles were conjugated to anti-mouse or anti-rabbit secondary antibody, AURION) diluted in incubation buffer. The primary antibodies were rabbit anti-1a antiserum (1:50), mouse-anti dsRNA monoclonal antibody J2 (1:50). Secondary antibodies were diluted at 1:20. Sections were counterstained with uranyl acetate (10 minutes) and lead citrate (3 minutes) and observed under a JEOL JEM 1400 TEM at 80KV at the Virginia-Maryland College of Veterinary Medicine.

### Immunofluorescence microscopy

Yeast cells were harvested and fixed with 4% formaldehyde for 30 minutes. To prepare spheroplasts, the cell wall was removed by lyticase. After permeabilization with 0.1% Triton X-100 for 15 minutes, the spheroplasts were incubated with primary antibodies (1:100 dilution) overnight at 4°C followed by incubation with secondary antibodies (1:100 dilution) for 1 hour at 37°C. Finally, the nucleus was stained with DAPI (Vector laboratories) for 10 minutes. Samples were observed using a Zeiss epifluorescence microscope (Observer.Z1) at the Fralin microscopy facility, VT.

### Measurement of yeast nuclear membrane perimeters

Measurements were performed with ImageJ software. Briefly, the scale was set based on the scale bar in images. The color threshold was adjusted to allow the spot to fit the nucleus perfectly and adding the target spot to the ROI (Region of Interest) manager by using the wand (tracing) tool. The perimeter was measured by performing the “measure” in the ROI manager tool.

### Radiolabeling and analysis of lipids

The steady-state labeling of lipids with [2-^14^C] acetate was performed as described previously [91]. Briefly, equal number of cells (2.5 x 10^5^ cells/ml) were inoculated into SC-Ura-Leu with galactose as carbon source along with the [2-^14^C] acetate. The cells were grown to exponential phase (A_600nm_ = ~0.5) and harvested. Lipids were extracted [92] from the radiolabeled cells, and then separated by one-dimensional TLC for neutral lipids [93] or phospholipids [94]. The resolved lipids were visualized by phosphorimaging and quantified by ImageQuant software using a standard curve of [2-^14^C] acetate. The identity of radiolabeled lipids was confirmed by comparison with the migration of authentic standards visualized by staining with iodine vapor. The mol percentage of each neutral lipid or phospholipid was normalized to the total ^14^C-labeled chloroform fraction. Single factor ANOVA was used for statistical analysis of lipid differences between wt and mutants.

### BMV replication assay in *Nicotiana benthamiana*

Replication analysis of BMV, TRV, and TMV [95] in *N*. *benthamiana* was performed as previously reported [96]. *Arabidopsis thaliana AtPAH2*, *N*. *benthamiana NbPAH1A* and *NbPAH2A* were expressed in *N*. *benthamiana* leaves by agroinfiltration following a protocol described before [97]. The *AtPAH2* was cloned into pAG2p vector between an enhanced CaMV 35S promoter and a terminator [70]. The *NbPAH1A* and *NbPAH2A* were cloned into pPWHT vector between an enhanced CaMV 35S promoter and a terminator through gateway cloning. *N*. *benthamiana* leaves were first infiltrated with Agrobacteria (GV3101) harboring pAG2p-*AtPAH2*, pPWHT-*NbPAH1A* or pPWHT-*NbPAH2A* plasmid. Two days later, the same leaves were infiltrated with the mixed Agrobacteria cultures harboring plasmids that launch BMV RNA 1, 2, and 3, or TRV1 and 2, or TMV. The infiltrated leaves were harvested 3 days post viral inoculation. Total RNA was extracted following the hot phenol method. Viral RNA accumulation was analyzed by Northern blotting with BMV-, TRV-, or TMV-specific probes as described before [70].

## Supporting information

S1 Fig BMV replication protein 1a localizes to the BMV-induced spherular VRCs in cells lacking *PAH1*. Immunogold labeling of BMV 1a in wt (A), *pah1*Δ *nem1*Δ (B) or *pah1*Δ *dgk1*Δ (C) cells in the presence of BMV replication. Anti-1a antiserum was used as a primary antibody and a 15-nm gold particle-conjugated anti-rabbit antibody was used as a secondary antibody. Micrographs at a higher magnification (A-1, B-1, B-2, C-1 and C-2) are also shown. (D) Percentage of gold particles localized in or near spherular structures in wt, *pah1*Δ *nem1*Δ or *pah1*Δ *dgk1*Δ cells. The total number of gold particles counted in each strain is also included.
(TIF)

S2 Fig The dramatically proliferated nuclear ER membrane in *pah1*Δ *nem1*Δ cells during BMV replication. (A) and (B) Micrographs showing BMV-replicating *pah1*Δ *nem1*Δ cells with proliferated membranes. The micrographs of boxed areas at a higher magnification are shown in A-1, B-1, and B-2. Arrows indicate the dramatically proliferated membranes.
(TIF)
