# Supplementary figures and images for: Host Pah1p phosphatidate phosphatase limits viral replication by regulating phospholipid synthesis
Source: PLoS Pathog. 2018 Apr 12;14(4):e1006988. doi: 10.1371/journal.ppat.1006988 (PMC5916857; doi:10.1371/journal.ppat.1006988)

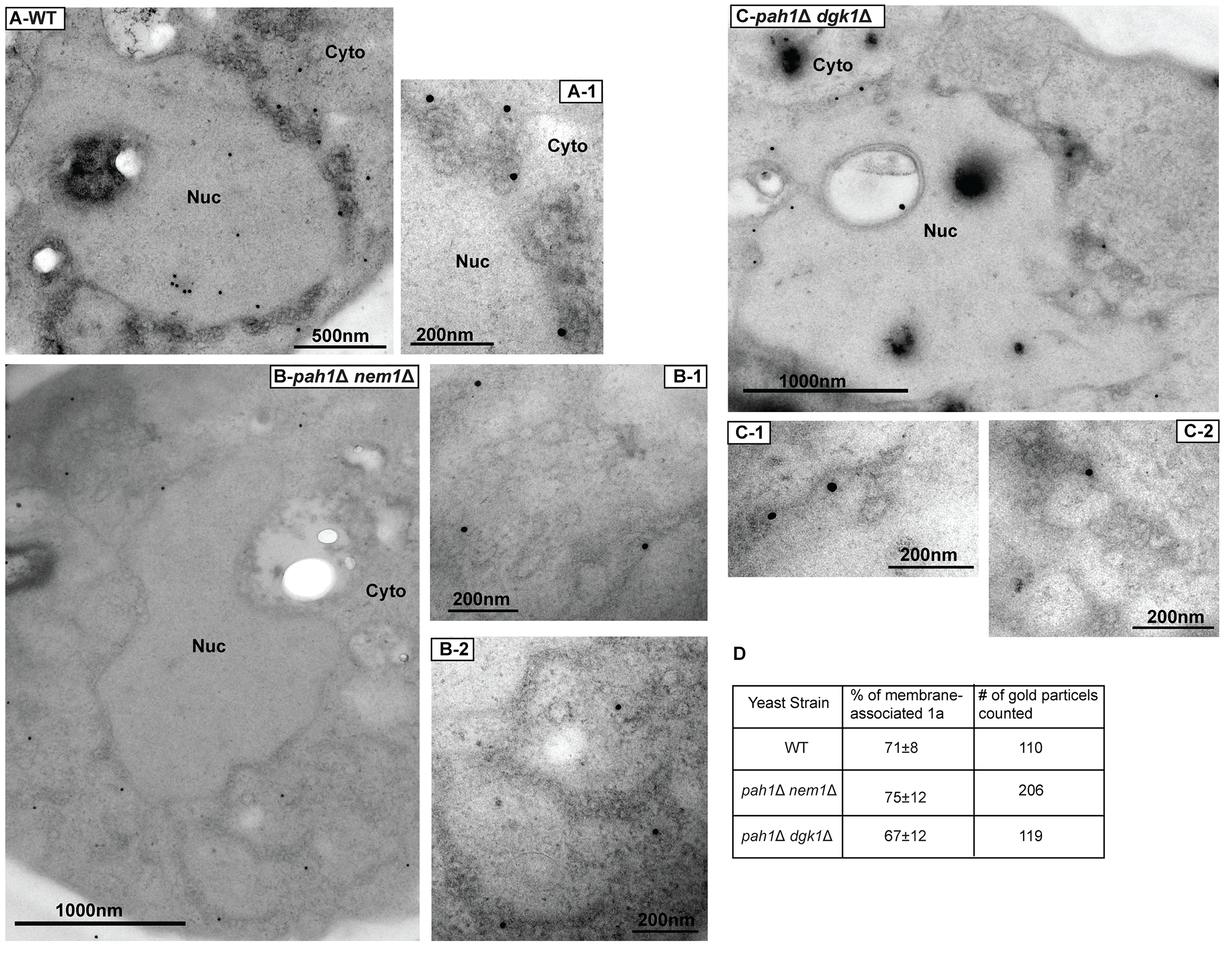

Supplement: S1 Fig — Immunogold labeling of BMV 1a in wt (A), pah1Δ nem1Δ (B) or pah1Δ dgk1Δ (C) cells in the presence of BMV replication. Anti-1a antiserum was used as a primary antibody and a 15-nm gold particle-conjugated anti-rabbit antibody was used as a secondary antibody. Micrographs at a higher magnification (A-1, B-1, B-2, C-1 and C-2) are also shown. (D) Percentage of gold particles localized in or near spherular structures in wt, pah1Δ nem1Δ or pah1Δ dgk1Δ cells. The total number of gold particles counted in each strain is also included. (TIF) [file ppat.1006988.s001.tif]

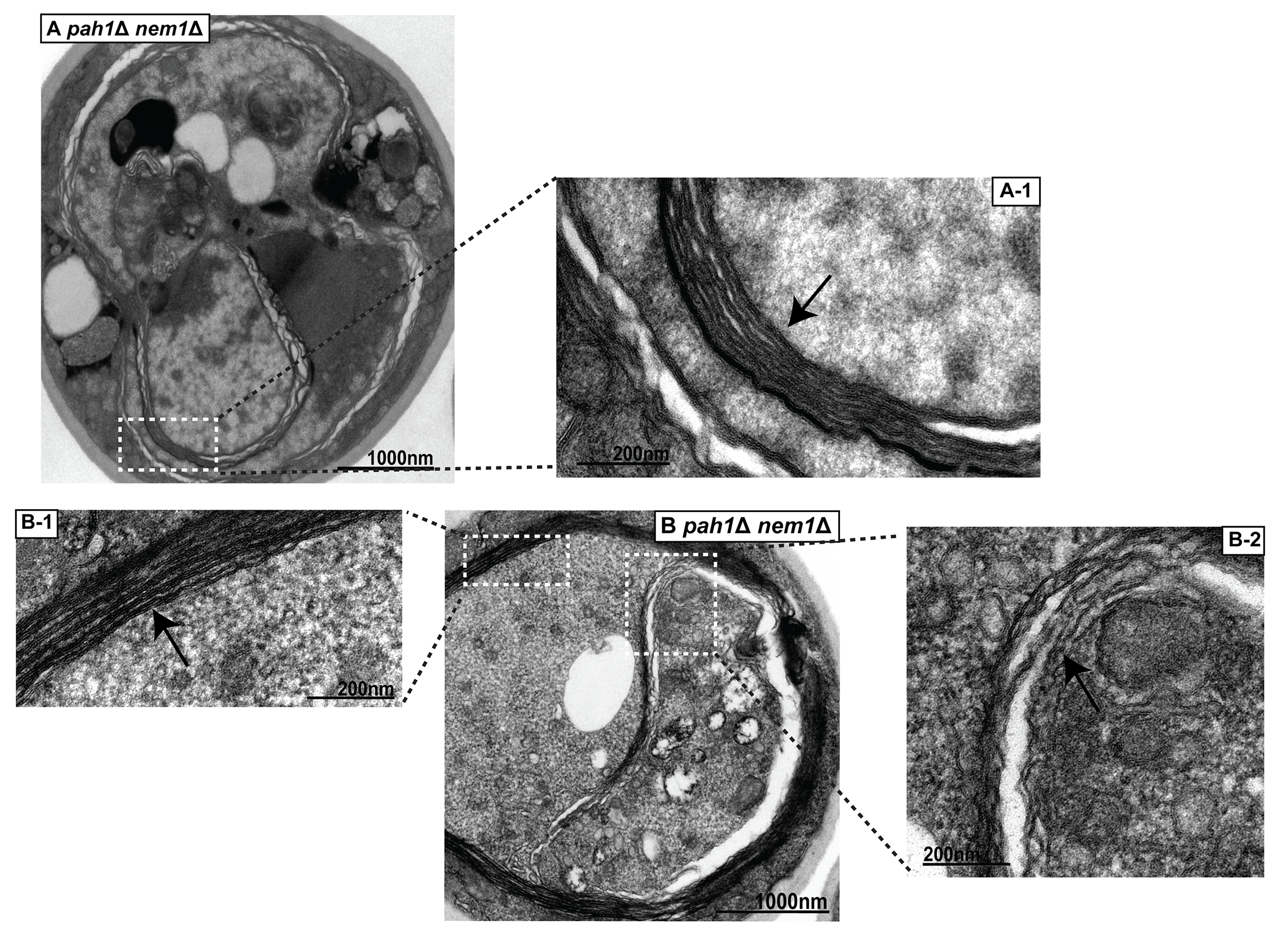

Supplement: S2 Fig — (A) and (B) Micrographs showing BMV-replicating pah1Δ nem1Δ cells with proliferated membranes. The micrographs of boxed areas at a higher magnification are shown in A-1, B-1, and B-2. Arrows indicate the dramatically proliferated membranes. (TIF) [file ppat.1006988.s002.tif]
